# Engineering nonlinear epileptic biomarkers using deep learning and Benford’s law

**DOI:** 10.1038/s41598-022-09429-w

**Published:** 2022-03-30

**Authors:** Joseph Caffarini, Klevest Gjini, Brinda Sevak, Roger Waleffe, Mariel Kalkach-Aparicio, Melanie Boly, Aaron F. Struck

**Affiliations:** 1 Department of Neurology, University of Wisconsin-Madison, 1685 Highland Ave, Madison, WI 53705 USA; 2 Department of Psychiatry, University of Wisconsin-Madison, Madison, USA; 3 Department of Computer Science, University of Wisconsin-Madison, Madison, USA; 4 William S Middleton Memorial Veterans Hospital, Madison, WI USA

**Keywords:** Epilepsy, Computer science

## Abstract

In this study, we designed two deep neural networks to encode 16 features for early seizure detection in intracranial EEG and compared them and their frequency responses to 16 widely used engineered metrics to interpret their properties: epileptogenicity index (EI), phase locked high gamma (PLHG), time and frequency domain Cho Gaines distance (TDCG, FDCG), relative band powers, and log absolute band powers (from alpha, beta, theta, delta, low gamma, and high gamma bands). The deep learning models were pretrained for seizure identification on the time and frequency domains of 1 s, single-channel clips of 127 seizures (from 25 different subjects) using “leave-one-out” (LOO) cross validation. Each neural network extracted unique feature spaces that were interpreted using spectral power modulations before being used to train a Random Forest Classifier (RFC) for seizure identification. The Gini Importance of each feature was calculated from the pretrained RFC, enabling the most significant features (MSFs) for each task to be identified. The MSFs were extracted to train another RFC for UPenn and Mayo Clinic’s Seizure Detection Kaggle Challenge. They obtained an AUC score of 0.93, demonstrating a transferable method to identify and interpret biomarkers for seizure detection.

## Introduction

The gold-standard for seizure detection remains expert opinion. Dependence on human interpretation places limits on the accuracy and reliability of seizure detection in chronically implanted Intracranial Electroencephalography (iEEG) devices such as Neuropace^®^. The high volume of data makes manual iEEG review impractical. Automated seizure detection is a necessity to optimize treatment paradigms and clinical workflow for iEEG and to advance approaches to neuromodulation using techniques like seizure-forecasting and reinforcement learning to develop personalized medicine approaches to patient care.

Numerous iEEG analysis metrics have been proposed to detect seizures that are derived from the Fast Fourier Transform (FFT) of an iEEG segment, such as the band powers (alpha, beta, gamma, delta, and theta), epileptogenicity index (EI), and phase locked high gamma (PLHG). Analysis of band powers refers to the comparison between frequency bands in the power spectrum with different functional properties^1^. EI was developed by Bartolomei et al. to quantify the appearance of high frequency oscillations by averaging the energy ratio between high and low frequency bands over the lag in seizure onset time relative to a reference point. Since its development, EI has been incorporated into other algorithms to locate seizures both anatomically and temporally^2–4^. PLHG is a method for quantifying ictal phase-amplitude coupling by computing the average of the high frequency signal envelope weighted by the instantaneous phase lag between the low and high frequencies^5–8^. Like EI, it has also been a valuable biomarker in seizure localization^5,6^. We chose to use the alpha, beta, gamma, delta, and theta band powers with EI and PLHG as our “hand-crafted” biomarkers because they have been proven effective in identifying epileptogenic zones, are intuitively derived from predefined frequency bands, and have grounding in the current understanding of the neurophysiology underlying seizure generation^7–11^. The effectiveness and simplicity of the selected features make them benchmarks for comparing other engineered metrics for neurological applications, such as approximate entropy, sample entropy, and fuzzy entropy^9,11–14^.

In this study, we apply the observation that EEG recordings have been shown to follow Benford’s Law^15^ by briefly quantifying this conformity as a potential biomarker for seizures to provide a unique, nonlinear metric excluded by other power spectrum-based features. We quantified adherence to Benford’s law using the Euclidian Distance between the observed and ideal distributions (or Cho–Gaines distance) of measurements in both the time and frequency domains (TDCG, FDCG)^16^. Together, these metrics are advantageous because they are nonlinear metrics, independent of the FFT, and can be easily parallelized on a graphics processing unit (GPU).

In addition to engineered features, there is extensive work using deep neural networks (DNN) for seizure identification because they can be GPU accelerated, do not require explicitly designed features, and can abstract higher level details from training data. Such properties make them useful for real time perceptual applications. Convolutional neural networks are the most widely used neural network because of their successes in computer vision processes and have since been applied to scalp and intracranial EEG for seizure identification or other classification tasks^17–21^. The major flaw with CNNs and other neural networks, is that they are essentially “Black Box” approaches, making them difficult to approve for clinical use because clinicians cannot interpret how the network generates its predictions.

Many studies attempt to make their CNNs interpretable using a variety of methods. Schirrmeister et al. and Hossain et al. frequency modulated input signals to their neural networks to determine which power bands they learned. Another strategy is to fuse hand-crafted and deep learning features for seizure identification, so that the model will be guaranteed to be utilizing clinically relevant information^22^. A shortcoming of this method is that there is little examination of the deep learning features, essentially leaving them uninterpretable. The need for an interpretable feature space has led to the use of autoencoders because their feature embeddings can be compared to widely used hand-crafted features to determine clinical relevance for seizure identification^17,23–25^. Prominent features can also be selected using more interpretable machine learning techniques, such as linear regression or Random Forest Classifiers, to determine which embedded features contribute the most to the classification task. Despite these successes, autoencoder models often need to be fine-tuned to accomplish a particular task, ultimately negating the benefits of using a separate autoencoder network in the first place^24,25^.

It has been demonstrated that neural networks can be modeled to generate interpretable feature spaces either through comparison or fusion^22^, but there are a few common techniques that limit the transferability of pretrained models. For instance, the electrode configuration is commonly built into the neural network, which causes all input samples to be interpreted as 2D images and restricts the application of that model to recordings from the same hardware. This property also makes interpretation more difficult because the spatial relationships learned by the neural network are not as straightforward to examine as temporal or spectral features. In an analogous manner, the length of the input recordings also affects interpretability because the seizure recordings may need to be cropped for processing by the network, leading to data loss. In addition to the hurdles from the structure of the neural network, a common data augmentation technique is to expand the recordings using overlapping, sliding windows along the time axis. Expanding data in such a way is convenient and effective when training a model for making predictions within a specific subject. However, it leads to overfitting when applied to training models for many different subjects because it copies patient specific patterns across multiple sample points^7^.

Our goal was to address shortcomings in interpretability by designing deep learning models specifically for extracting seizure biomarkers from any input iEEG signal, regardless of recording length or electrode configuration, by designing them to process basic single channel, 1 s segments using a training algorithm that minimizes the repetition of time series data points. Each neural network was designed to resemble an autoencoder^26^, with an encoder sub network for learning feature representations and a classifier sub network in lieu of a decoder for classifying the encoded feature spaces. Classifiers were used instead of true autoencoders because the primary focus was the discovery of seizure biomarkers (that is, interpretable features that reliably predict seizures), and thus we designed our neural networks specifically to extract features characteristic of ictal activity. The data was divided into 1 s epochs because this would provide the greatest flexibility when using the model to annotate new clinical data while still being long enough to capture frequencies as low as 2 Hz. It also provides more accurate seizure localization for clinical applications and demonstrated efficacy for spike detection in iEEG recordings^27^. The training algorithm was designed to favor generalizability by employing a nested cross validation technique where a holdout set was generated by excluding all samples from a specific subject, and then a validation set was created by randomly selecting all samples from another eight subjects prior to training.

The overall result of this validation scheme is the ability to select models that learn features highly transferable (the seizure biomarkers) to unseen datasets. We also fuse and compare the deep learning features with “hand-crafted” metrics using Random Forest Classifiers (RFCs) to classify seizures because RFCs are highly interpretable models that allow us to compare the relative contributions of each feature while identifying specific biomarkers. We examine two neural networks for this purpose: one of them is a convolutional neural network designed to function as a series of discrete filters extracting features from the time domain, called the Time Domain Black Box (TDBB), and another, fully connected network for learning nonlinear relationships between power spectrum densities, called the Frequency Domain Black Box (FDBB), and a model using the engineered features (power spectrum, PLHG, EI, and Benford’s Law). The feature spaces from these models were compared to each other to demonstrate their accuracy in seizure detection and interpretability using a feature comparison paradigm.

## Results

### Encoder pretraining

**Figure 1:**
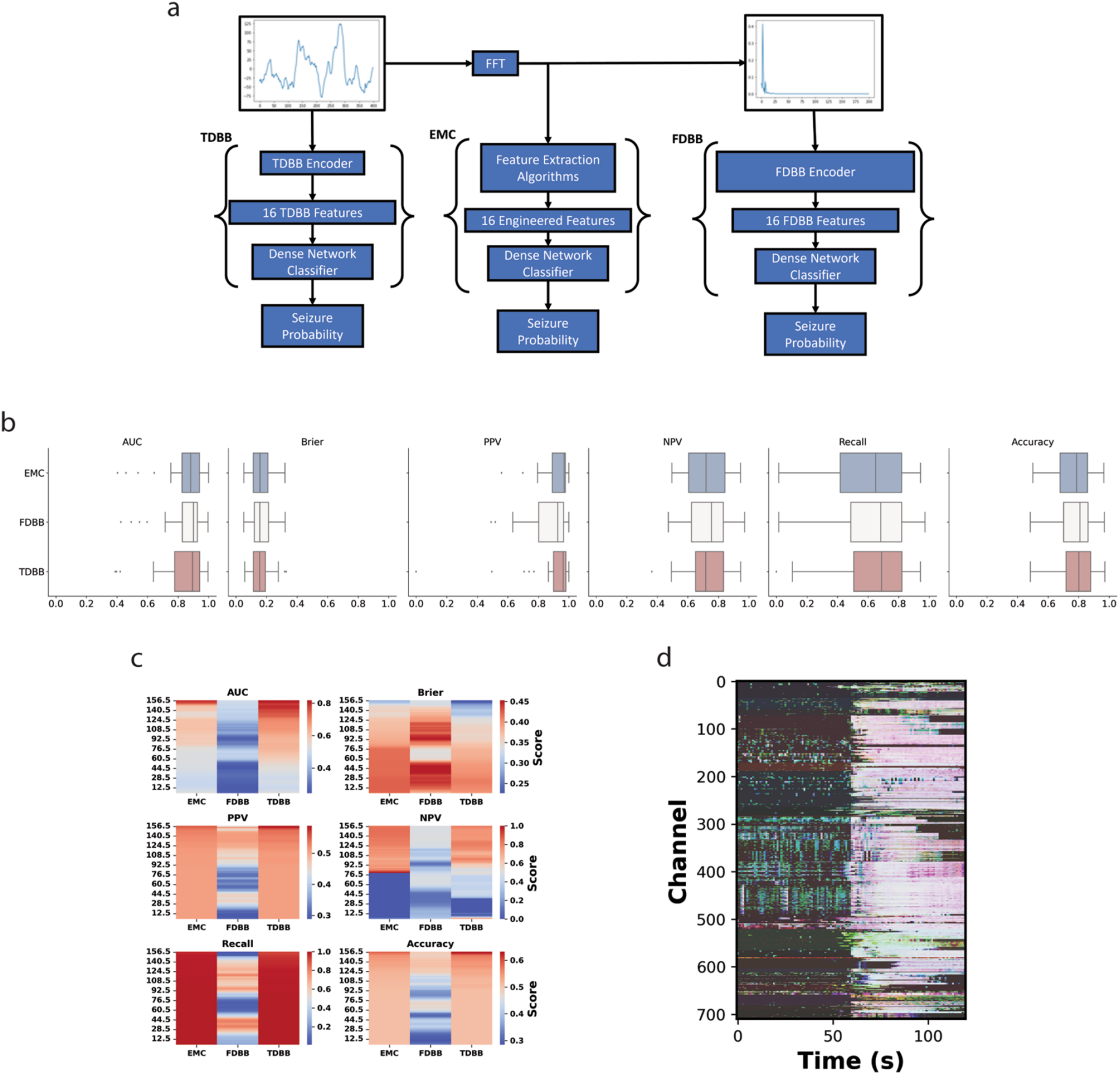
Neural network architectures, leave one out (LOO) pretraining, and evaluation. (**a**) Network schematic for each neural network. (**b**) LOO test set performance of each neural network on clinical (broadband) iEEG signals. (**c**) LOO test set performance on narrowband iEEG signals. (**d**) Visualization of model LOO test set predictions on iEEG data.

We used our collection of single channel, 1 s segments as a standardized dataset for pretraining our neural networks to extract features from any iEEG recording (Fig. 1a). We created a third network for classifying the hand-crafted metrics (called the engineered metrics classifier, or EMC) to verify that the extracted features have similar utility for seizure detection. This densely connected classifier subnetwork (as summarized in Table 3 and shown in Fig. 1a) was identical to the densely connected classifier subnetworks in each of the black box models (Fig. 1a). It was expected to exhibit the same performance on clinical recordings when given feature sets highlighting the same underlying phenomenon. However, we expected the performances to differ when given data filtered to a 5 Hz bandwidth (narrowband) iEEG signals, as each network should respond according to the patterns it learned from the data. The classification performance of each model was not the priority for this stage because we are more interested in evaluating interpretability, making the relative classification performances between each network on both the clinical and narrowband iEEG recordings much more significant to understanding the models. The “Model design and validation” section contains more information about neural network design, pretraining, and evaluation.

The three DNNs were trained with architecture described in Fig. 1a,b and Tables 1, 2 and 3 in the “Neural network design” section. The first two “black box NNs” use an encoder-classifier setup designed to automatically *encode* EEG data into learned features and then *classify* seizure probability based on these features. The EMC contains only a classifier, replacing the encoder with hand engineered features, and serves as a control for assessing the performance of the first two. Each model was pretrained on a “seizure detection” task and given a 1 s iEEG segment to predict the seizure probability. We used single channel segments to force the models to learn ictal-specific features and so they could be applied to recordings with different electrode configurations and lengths. After training, the black box neural networks were adept to extract features from iEEG data by computing the output of the encoder.

**Table 1:** Time domain black box (TDBB) encoder network parameters.

TDBB encoder	
Layers	Intermediate tensor shape
Input	(N, 1, 400)
**Conv1d**(1, 2, k = 27, p = 13), ReLU	(N, 2, 400)
**Conv1d**(2,4, k = 27, p = 13), ReLU, **batch normalization**	(N, 4, 400)
1D max pooling	(N, 4, 198)
**Conv1d**(4, 5, k = 13, p = 6), ReLU	(N, 5, 198)
**Conv1d**(5, 7,k = 13, p = 6), ReLU, **batch normalization**	(N, 7, 198)
1D max pooling	(N, 7, 97)
**Conv1d**(7, 9, k = 7, p = 3), ReLU	(N, 9, 97)
**Conv1d**(9, 10, k = 7, p = 3), ReLU	(N, 10, 97)
**Conv1d**(10, 11, k = 7, p = 3), ReLU, **batch normalization**	(N, 11, 97)
1D max pooling	(N, 11, 46)
**Conv1d**(11, 12, k = 5, p = 2), ReLU	(N, 12, 46)
**Conv1d**(12, 13, k = 5, p = 2), ReLU	(N, 13, 46)
**Conv1d**(13, 14, k = 5, p = 2), ReLU, **batch normalization**	(N, 14, 46)
1D max pooling	(N, 14, 21)
**Conv1d**(14, 15, k = 3, p = 1), ReLU, **batch normalization**	(N, 15, 21)
1D max pooling	(N, 15, 8)
**Conv1d**(15, 16, k = 8, p = 0), ReLU	(N, 16, 1)

The convolutional layers were designed to act as a series of discrete filters with trainable parameters. N is the batch number of the input tensor.

Trainable layers are given in bold.

**Table 2:** Frequency domain black box (FDBB) encoder network parameters.

FDBB encoder	
Layers	Intermediate tensor shape
Input, **batch normalization**, 75% dropout	(N, 200)
**Linear**(200,100), leaky ReLU, 75% dropout	(N, 100)
**Linear**(100,50), leaky ReLU, 75% dropout	(N, 50)
**Linear**(50,16), leaky ReLU	(N, 16)

The fully connected subnetwork was designed to learn how to derive relationships between the power densities of each frequency band. N is the batch number of the input tensor.

Trainable layers are given in bold.

**Table 3:** Classifier subnetwork parameters.

Classifier subnetwork	
Layers	Intermediate tensor shape
Input, **batch normalization**, dropout	(N, 16)
**Linear**(16,14), leaky ReLU	(N, 14)
**Linear**(14,12), leaky ReLU	(N, 12)
**Linear**(12,10), leaky ReLU	(N, 10)
**Linear**(10,8), leaky ReLU, **batch normalization**, dropout	(N, 8)
**Linear**(8,6), leaky ReLU	(N, 6)
**Linear**(6,4), Leaky ReLU	(N, 4)
**Linear**(4,2), sigmoid	(N, 2)
**Linear**(2,1), sigmoid	(N, 1)

This classifier subnetwork is used in all neural networks with the same parameters. Dropout was adjusted to prevent overfitting for each. N is the batch number of the input tensor.

Trainable layers are given in bold.

If the information captured by the DNNs was characteristic of seizure activity, then we expected all models to exhibit similar performance on the “seizure identification” task because they have identical classification sub networks and are classifying the same data. As shown in Fig. 1b, the black box networks performed about the same as the EMC network on the pretraining task, with the FDBB scoring a slightly higher AUC score than the TDBB, and slightly worse than the EMC (AUC 0.836 ± 0.31 vs 0.823 ± 0.35 and 0.837 ± 0.31, respectively), all the models performed within the range of state-of-the-art accuracies presented by other 1D convolutional neural networks^28^. We then gained deeper insight into the properties of each network by bandpass filtering the test folds with 5 Hz windows at regular 2 Hz intervals, as explained in the “Neural network pretraining and evaluation” section of the “Methods”; the results are shown in Fig. 1c. The fact that the TDBB and EMC both demonstrated clear positive correlations between low gamma and high gamma activity and accuracy suggests that they learned to correctly associate these frequencies to seizures^29,30^. Conversely, the FDBB demonstrated strong negative correlations between frequencies in the delta, theta, alpha, and beta bands, suggesting that it learned to correctly identify which bands are not indicative of seizure activity. The discrepancies between the broad and narrow band scores demonstrate how the models learned to associate information from different frequency bins to make their classifications. The similar performance between all neural networks and their sensitivity to seizure specific frequency ranges indicates that the black box feature spaces contain information that is just as relevant to seizure activity as the engineered metrics ensemble, and thus we expect that the deep learning features could potentially be extracted from other seizure recordings for classification, just as hand crafted metrics are currently used in clinical applications. Visualizations of the LOO test set predictions are included in Fig. 1d. The test folds are shown in the Supplementary Material in the same order as the rows in Fig. 1d.

### Feature interpretation and selection

**Figure 2:**
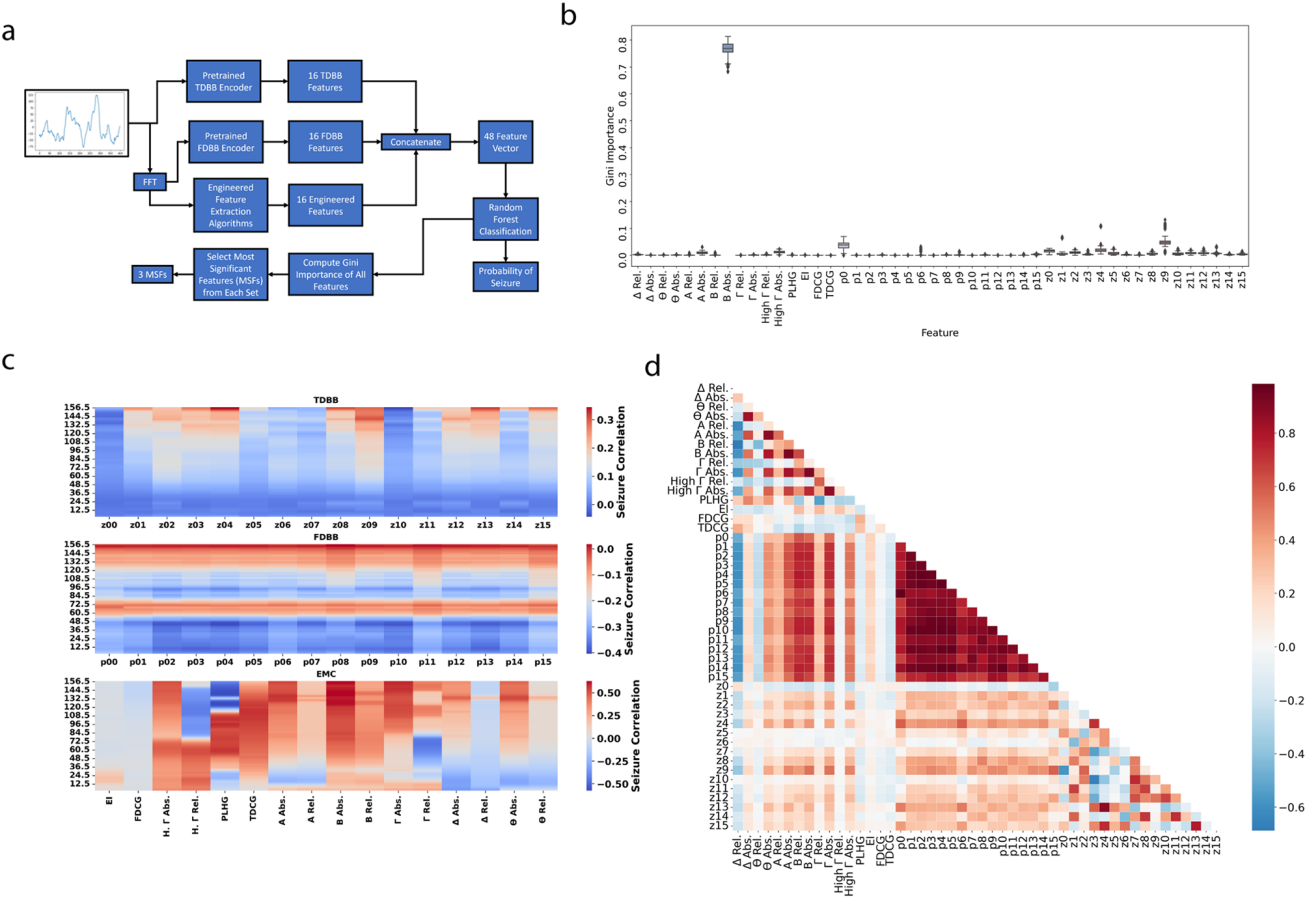
Process for identifying and interpreting significant features during pretraining using random forest. (**a**) Process using Random Forest Classifiers (RFCs) to identify the most significant features (MSFs). (**b**) Relative Gini Importance of all features. (**c**) Frequency response of encoded feature spaces. (**d**) Correlation between feature spaces.

The pretraining stage demonstrated that each DNN can extract biomarkers indicative of seizure activity and demonstrated that these classifiers exhibited complementary frequency responses. Once this was demonstrated, they could be applied to study the significance and interpretability of the extracted features from the pretrained models and see if their complementary performances translate to complementary feature sets. To achieve this goal, a Random Forest Classifier (RFC), was created using an ensemble feature set made by concatenating the features from the TDBB, FDBB, and EMC. We chose to use an RFC because it is a highly interpretable machine learning algorithm that allowed the relative importance of all features to be evaluated and compared for seizure identification; the results are summarized in Fig. 2b. In “Feature selection” section details the steps performed for feature space analysis.

We also used the same frequency modulation setup from the “Encoder pretraining” step to establish the relationships between each deep learning feature, signal frequency, and seizure activity; the results are included in Fig. 2c. They describe how the frequency responses of each encoder space affect its correlation to seizure activity and allow us to explain the associations uncovered by the RFC. We saw the highest positive correlation to seizure activity to be with beta log absolute band power (or beta power), with similar correlational patterns between the FDBB features and TDBB features, and we expected the RFC to also reflect these results.

After training the RFCs on all 48 of the features and computing the Gini Importance for each, we determined that the most significant features (MSFs), in order, were the beta power, followed by p0, and then z9, where p0 is the first FDBB feature and z9 is the tenth TDBB feature. This observation agrees with the analysis of Fig. 2c, as the beta power was the only feature with a strong positive correlation to seizures at all frequencies. The other two features, p0 and z9, as shown in Fig. 2d, appear to be composites of the alpha, beta, low gamma, and high gamma band powers. Additionally, they were the only unrelated features between the TDBB and FDBB ensembles containing the same power band associations, indicating that they are capturing related, but mathematically distinct properties of the iEEG signal. Surprisingly, little to no correlation was seen between the Black Box feature sets and nonlinear features like EI and PLHG, indicating that the models learned a different, unspecified relationship between band powers. The correlation of the DNN feature sets with each other and the log absolute band powers illustrates that they generated nonlinear combinations of clinically relevant features, potentially forming new biomarkers for use in clinical applications.

### Deployment on Kaggle dataset

**Figure 3:**
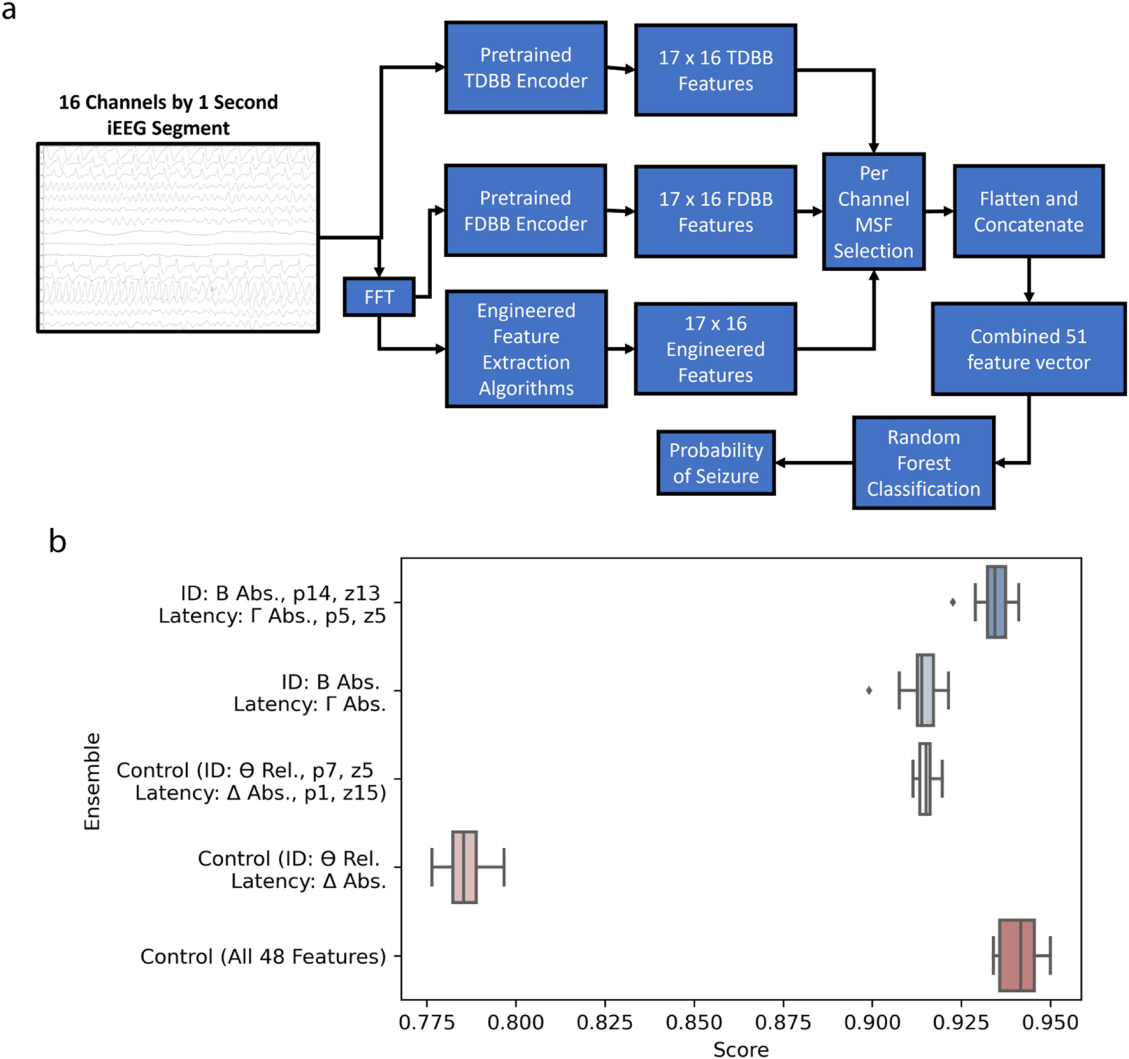
Kaggle test scores using most and least significant feature (MSF and LSF) ensembles. (**a**) Kaggle contest training process on a hypothetical canine data point. (**b**) Kaggle Autograder Scores on the test set using feature ensembles with varying importance.

After the MSFs were identified, we evaluated their transferability by using them to identify seizures on a completely unseen dataset. We tested the flexibility of our approach using the UPenn-Mayo Kaggle Seizure Detection Challenge dataset because it contains a mix of human and canine iEEG data with many different electrode configurations (that are each 1 s long), and thus allowed us to demonstrate that extracting features with pretrained neural networks as feature encoders is a highly versatile application of deep learning in seizure detection. We extracted the features using deep learning from each channel, then combined them together for classification using RFCs. We created several ensembles: 3 MSFs (beta power, p0, and z9), 1 MSF (beta power), 3 least significant features (or “LSFs”: theta relative band power, p7, z5), and the single LSF (theta relative band power), and all 48 features to verify that the most important features could be selected and utilized as seizure biomarkers without significantly hindering performance. The MSFs and LSFs for latency are shown in Fig. 3b. We also compared the RFC classifiers with a purely deep learning approach to demonstrate that spatial information does not need to be considered by the neural networks, and instead can be included downstream by aggregating the features from each channel. The TDBB model was modified to work with multiple channels so it could be used as the purely deep learning approach by multiplying the number of filters at each stage of the network by the number of electrodes in the recording to process all of them simultaneously. It obtained a maximum test AUC score of 0.87. The various ensembles taken from the encoded features outperformed the purely deep learning model by achieving a maximum AUC score of 0.93, demonstrating that our proposed method results in composite features that are more flexible and transferable than traditional deep learning approaches. It also demonstrates that more meaning is extracted from the underlying iEEG recording if features are first extracted from the time series, and then aggregated into spatial relationships later, rather than attempting to do both at the same time with a neural network. The Kaggle test scores for each of the MSFs, LSFs, and all features as the controls are included in Fig. 3, and an in-depth description of the Kaggle Challenge is found in the “Deployment” section.

## Discussion

We have shown that neural networks can be designed and trained to learn novel biomarkers for seizure identification that can be interpreted by comparing them to hand crafted features and evaluating their sensitivities to the frequency composition of the underlying iEEG recording. The encoder—classifier architecture allowed for the development of feature embeddings that could be aggregated into spatial relationships after extraction to make subject-specific classifications, and classifications specific to seizure onset type, such as rhythmic theta activity versus low amplitude fast frequencies. The TDBB and FDBB both were able to encode latent spaces capable of achieving state of the art performance in seizure identification. The pretrained encoders from both DNNs generated feature spaces highly transferable to new datasets. The MSFs from these pretrained models were selected and applied to the Kaggle Challenge, where they achieved an AUC score 0.93 using MSFs, and 0.95 when using all features. In the former case, our MSF ensemble was significantly smaller than the features used by other, higher-ranking submissions while using a simpler RFC model (30 estimators vs 3000 estimators) to generate predictions. These differences made our MSF ensemble more computationally efficient to train on longer recordings without significantly hurting performance. Additionally, our models were able to achieve scores within the ranges of those reported by other state of the art methods while using a smaller, more interpretable feature set and shorter time window, making them more attractive for implanted iEEG devices with limited computational power^21,31,32^. The strong performance on the Kaggle competition demonstrates that MSFs identified during pretraining are transferable to new datasets, that DNNs are capable of extracting generalizable features of seizures between subjects and electrodes from iEEG recordings, and that the spatial information in the signals can be aggregated downstream of feature extraction in a task-specific manner. Even more surprising was that the Kaggle dataset was montaged differently than the pretraining data (common average reference vs common reference), but did not hinder performance, implying that any differences in feature properties from montaging can be compensated for by other machine learning models.

It is important to note that the MSFs from DNNs change depending on the size of the dataset and cross-validation method (LOO vs by channel, or sample) used to pretrain the models. This finding suggests that to accurately transfer features, extra care is required to prevent overfitting and data leakage during pretraining. It also suggests that intrasubject neural networks are likely to learn signal properties unique to a specific patient. As a general practice to make a generalizable seizure detection algorithm, it is critical that the training and validation datasets use completely different subjects, not just different seizures, or channels from the same subject. It is common to overfit to patient specific seizure factors that limit transferability and over-estimate the accuracy of a model.

There are many other seizure identification experiments using neural networks and engineered features^28,33,34^, but direct comparison to many of the models is difficult because of inconsistencies between the performance metrics chosen, how the models are trained, how the scores are reported (maximum achieved vs average score), and the composition of the dataset. Data composition matters because most projects report accuracy, which has recently been shown to be inconsistent due to its dependency on the data composition and the model architecture^21,35^. Regardless of the metric, performance can be inflated if (i) there is a large class imbalance between ictal and preictal data points, (ii) data leakage between validation or testing groups exists, or (iii) the validation scores are reported from cross validation instead of a separate holdout set. These inflation-fostering conditions are difficult to grasp without source code. Often score reporting lacks consistency in describing whether the result refers to the achievement of a maximum or an average score. This could imply the reporting of outliers rather than expected performance. To overcome the issues identified as obstacles in model comparison, our models were compared to others that completed similar tasks with some comparable metrics. A useful source for assessing performance was the UPenn and Mayo Clinic’s Seizure Detection Challenge for reasons outlined in the “Kaggle challenge” section. We can conclude that our proposed method of designing and training neural networks specifically for biomarker discovery was able to extract seizure biomarkers across different subjects, as evidenced by its ability to obtain state of the art performance in a simple identification task.

Our method also generated features that could be interpreted through the analysis of the frequency sensitivities of all models and their features, as well as through the correlation between our selected hand-crafted features and the learned features. The best features from our Black Box encoders can be identified and selected using Gini Importance with minimal preprocessing for use in other machine learning tasks, and their ability to be generated once every second makes them potentially useful for seizure forecasting. Notably, we found that the simplest features, such as the beta and low gamma log absolute band powers, are the most important, and may be foundational to engineer novel epileptic biomarkers for early seizure detection. This suggests that a similar experiment could be designed using a neural network to learn composite biomarkers from the hand-crafted features.

The findings of this study can be used to encourage future work in signal processing, seizure forecasting, and seizure localization as the flexibility of this model lends it well to sequence analysis. The ability to evaluate the frequency response of the models, at both the output and feature embedding levels, means that the relative sensitivities to unwanted frequency bands could potentially be identified and removed from the signal to enhance seizure detection. Additionally, the fact that feature spaces and model predictions summarize 1 s segments and can be computed on a GPU means they could be extracted in real time applications for use in sequence learning models for seizure forecasting. Analogously, the features could be extracted from all channels separately allowing them to be used to determine the seizure onset zone from iEEG recordings. Further, the ensemble with a sparse feature set based on generalizable biomarkers is easy to train on patient-specific and electrode-specific data to optimize seizure detection on an individual patient, with minimal need for experts to manually mark seizures.

## Methods

### Subjects, standard IEEG dataset, and formatting

Approval was obtained from the University of Wisconsin Institutional Review Board, informed consent was obtained from all human subjects prior to participation. All methods were conducted in accordance with the Declaration of Helsinki. After approval of the respective IRBs, the raw EEGs from 25 pre-surgical epilepsy patients were visually inspected. The seizure onset time and channels involved with seizure onset were marked and confirmed by a board-certified neurophysiologist (Boly M). Only channels involved at seizure onset were used for further analysis.

The selected dataset consisted of 710 channels taken from 127 different seizures across 25 individuals: 13 seizures from five patients were from the University of Wisconsin; 82 seizures from 13 patients were from the Epilepsiae Database^36^; 32 seizures from seven individuals were provided by Mayo Clinic^37^. Data from the Epilepsiae database was prefiltered with a 50 Hz notch filter, and data from Mayo Clinic and the University of Wisconsin were prefiltered with a 60 Hz notch filter. Each recording was shown in a common reference montage and filtered with an anti-aliasing lowpass filter (passband edge of 180 Hz) before being resampled to 400 Hz. Then the signals were subjected to a zero-phase finite impulse response (FIR) high pass filter with passband edge of 1 Hz. Each channel containing seizures was selected and cropped to two minutes in length, with seizure onset being aligned 1 min into the segment. The preictal labels were assigned to every point before seizure onset, and the ictal labels were assigned to every point at and after seizure onset, creating an even amount of preictal and ictal data points. Overall, there were 15,240 s of data, consisting of 50% preictal and 50% ictal/post-ictal points, where each point is a 1 s clip, and an average of 28 electrodes per patient. Overall, there were 295 depth electrodes (41.54%), 109 strip electrodes (15.35%), and 306 grid electrodes (43.09%). Figures of the pretraining dataset are included in the Supplementary Material and are divided by subject, along with an image illustrating the brain regions recorded in the pretraining data.

The pretraining and feature selection data was formatted to be non-overlapping, single channel, 1 s segments. Samples were non overlapping to avoid overfitting patient specific seizure patterns in the training data. The data was split into 1 s segments to encourage neural networks to learn high frequency features while enabling more precise seizure localization. Each recording was divided into single channels to allow the models to be applied with flexibility to any type of electrode configuration. Both formatting methods also expanded the dataset to include more training samples.

### Computing engineered features

The hand-crafted features are mostly derived from the power spectrum of each iEEG recording because they have been proven as effective biomarkers and can be compared to the black box features to illustrate the signal properties learned by the black box models. The exceptions are the TDCG and FDCG distances, which were included to provide a few nonlinear metrics unrelated to the FFT that may have clinical utility. They also provided a way to evaluate if the neural networks could learn to extract trends unrelated to the power spectrum of the signal. All features are described in detail in “Computing digit distributions and Cho Gaines distance” to “Phase locked high gamma (PLHG)” sections.

#### Computing digit distributions and Cho Gaines distance

The entire dataset was broken into 1 s epochs, and the power spectrum of each epoch was computed using the magnitude of the FFT. The first nonzero digits were counted and normalized to generate a probability distribution over the extracted digits. The leading nonzero digits were extracted from each time point using the following element-wise formula:

1 $$l = \left| {\frac{{\left| {\text{e}} \right|}}{{10^{{\left| {{\text{log}}_{{10}} \left( {\left| {\text{e}} \right|} \right)} \right|}} }}} \right|,$$

where e represents an arbitrary sized tensor. The results were set to zero whenever the function was undefined, i.e., where e = 0. The specific digits between one and nine were counted and normalized to generate a probability distribution over the extracted digits within the window. The Cho-Gaines Distance (a.k.a. Euclidian Distance^16^) was used to compare observed probability distributions (Eq. (3)), with expected probability distributions as calculated using the formula from Benford’s 1937 work^38^, as shown in Eq. (2).

2 $${\mathrm{P}}_{\mathrm{d}}={\mathrm{log}}_{10}\left(\frac{\mathrm{d}+1}{\mathrm{d}}\right),$$

where d is a digit from {1,2,3,4,5,6,7,8,9}.

3 $$\mathrm{D }= \sqrt{N\times \sum_{i=1}^{9}{({e}_{i}-{o}_{i})}^{2}},$$

where N is the EEG sample rate, e_i_ is the expected frequency that i is the first digit, o_i_ is the observed frequency where i is the first digit.

#### Band powers

The delta (2 to 4 Hz), theta (4 to 8 Hz), alpha (8 to 12 Hz), beta (12 to 30 Hz), low gamma (30 to 80 Hz), and high gamma (80 to 150 Hz) band powers were computed by using Welch’s method^1,10,39^. Band powers were then normalized by the total signal power to create the relative band powers, and the log of the absolute band powers were also used as features. The DC component (f = 0 Hz) was dropped before computing relative band powers.

#### Epileptogenicity index (EI)

The EI consists of averaging the energy ratio (ER) over the delay in seizure onset from a specified reference point, but our use of 1 s segments simplify the calculation by reducing all time intervals to one, as shown in Eqs. (4) and (5)^10^. The ER was calculated using the ratio band powers from the alpha, beta, theta, and gamma bands, within each 1 s segment as shown in Eq. (4)^10^:

4 $$\mathrm{ER}= \frac{{\mathrm{P}}_{\upbeta }+{\mathrm{P}}_{\upgamma }+{\mathrm{P}}_{{\upgamma }^{\mathrm{^{\prime}}}}}{{P}_{\theta }+{P}_{\alpha }},$$

where P are the relative band powers of the α, β, γ, γ’, and θ are the alpha, beta, low gamma, high gamma, and theta bands, respectively. The ER is then used for computing EI is given in Eq. (5)^10^:

5 $$\mathrm{EI}= \frac{\sum_{n={N}_{d}}^{{N}_{d}+ 1}ER[n]}{{{N}_{d} - N}_{0}+ 1} = \frac{ER}{{{N}_{d} - N}_{0}+ 1},$$

where N_d_ is the delay in sample points from the reference point, N_0_. N_d_ is typically determined by setting an amplitude threshold on the ER and recording the sample point where this threshold is tripped. Because machine learning models can independently learn the ER threshold, we could ignore N_d_ and consider N_0_ as the start of each data point, allowing for us to further simplify the formula to Eq. (4).

#### Phase locked high gamma (PLHG)

The formula for computing PLHG is shown in Eq. (6)^8^:

6 $$\mathrm{PLHG }= \left| \frac{1}{n}\sum_{1}^{n}{A}_{HFO}\times {\mathrm{e}}^{{\Phi }_{LF}- {\Phi }_{HFO}}\right|,$$

where A_HFO_ is the instantaneous signal envelope of the high frequency oscillations, and ${\Phi }_{LF}- {\Phi }_{HFO}$ is the difference between the low and high frequency instantaneous phases. LF in this case means from the theta, alpha, and beta bands, where the HFO means from the low gamma and high gamma bands.

### Model design and validation

We designed our neural networks to function essentially as mathematical formulas for computing biomarkers. Their performance on the pretraining dataset was not the chief concern at this stage. Instead, we were concerned with their performance relative to the set of hand-crafted features because this would demonstrate if they were learning features with information comparable to the hand-crafted set.

#### Neural network design

The TDBB was designed as a convolutional neural network to imitate a series of discrete filters with trainable kernel parameters to progressively select for higher frequency bands in each layer. The FDBB was designed using fully connected layers because most power spectrum-based metrics are composed from nonlinear combinations of frequency bins, and thus could be mimicked using a fully connected network with an exponential activation function. These approaches were developed because they give the model the freedom to learn its own associations that may be more applicable to seizure identification than hand-crafted features.

In total, three neural networks were designed to learn from 1 s segments of iEEG data: the TDBB, FDBB, and a fully connected network for classifying engineered metrics (EMC). Each DNN had distinct encoder and classifier sub networks to allow the networks to be used in dimensionality reduction. The classifier sub network was the same in all three models to allow for the comparison between different feature spaces. The output of the encoder sub networks was used for feature analysis and downstream classification tasks. The dropout in the classifier sub network was 75% for FDBB, 90% for the TDBB, and 40% for the EMC. All networks were designed to work on any arbitrary number of electrodes by changing the input channels parameter. This allowed the same network architecture to be applicable to more datasets, such as the 2015 Kaggle competition “UPenn and Mayo Clinic’s Seizure Detection Challenge”, Kaggle contest^40–43^. The flow diagram depicting all architectures of all three models is included in Fig. 1a, and the specific parameters of the encoder and classifier subnetworks are included in Tables 1, 2 and 3.

#### Neural network pretraining and evaluation

All three neural networks were trained using leave-one-out (LOO) cross validation across 25 subjects, where all the data points from one patient were withheld for training. For each fold, models were trained using stochastic gradient descent, Adam optimizer with a learning rate of 0.001, and binary cross entropy loss until there was no improvement in the validation loss for ten epochs (or 200 epochs were reached, whichever occurred first). Training and validation sets were also separated by subject, with eight training subjects being assigned at random to the validation set. The training set was shuffled to ensure different validation and training sets for each fold. The AUC, Brier, PPV, NPV, Recall, and Accuracy were all recorded for each test set, and the model with the best overall scores was selected to attempt the Upenn-Mayo Kaggle challenge. Cross validation was conducted with the same random seed, allowing the data points used in each fold to be identifiable and consistent across models.

The sensitivities of the output probabilities, performances, and feature spaces relative to signal frequency bands were calculated following a process from Schirrmiester et al. and Hossain et al. to evaluate which signal components were being learned by the neural networks. This was done by first band pass filtering the training iEEG recordings using 5 Hz frequency bins with a bandpass biquad filter using the median frequency as the resonant frequency, with the median frequency being incremented at 2 Hz intervals from 2 to 155 Hz. Filtered signals were then minmax normalized and rescaled to be the same amplitude as the negative training sample class. This was done because the models are sensitive to amplitude and need the input to be the same magnitude as the training set. The resulting predictions, scores, and feature spaces were all extracted from the model outputs, and their point biserial correlation with seizure activity was calculated to determine the sensitivities of each neural network for each frequency band. The classification performance with respect to the resonant frequencies for each model was also recorded using the same method.

### Feature selection

Once our black box models were trained, and they achieved a classification performance comparable to the EMC classifier, their encoder layers were frozen and used to extract features from each holdout fold of the pretraining data. These features were then used to train an RFC so that the relative contributions of each feature could be interpreted. The pretrained model from each LOO fold was saved separately so that the model being tested by the holdout set could be selected for the feature selection stage, preventing data leakage.

#### Feature analysis

For each fold, the test subject and associated pretrained model were selected to extract the FDBB and TDBB feature spaces, preventing data leakage during RFC training. The feature spaces were then analyzed using Gini Importance and a correlation analysis. Gini Importance is a computationally efficient splitting criterion used in RFCs that splits samples into nodes minimizing the impurity of each node during fitting, making it the most efficient way of evaluating features when using RFCs^44^. The correlation matrix between all feature combinations was computed to illustrate potential relationships between the DNNs and engineered metrics, and enabled us to determine if the DNN feature spaces were related to the engineered metrics.

#### Feature ensembles

As summarized in Steps 2 and 3 in Fig. 2a, leave-one-out (LOO) cross validation of a Random Forest Model across 25 subjects was used to generate interpretable models using all features for both seizure identification and latency determination tasks. The latency task is defined by the UPenn-Mayo Kaggle challenge and consists of identifying ictal segments from the first 15 s of the seizure as the positive group (12.5% of samples)^40–43^. The dataset was expanded in the pretraining stage by splitting all channels for each subject and using a 1 s sliding window with 50% overlap to generate additional frames, as demonstrated in the winning Kaggle Submission. For the remaining 24 subjects within each LOO fold, an RFC (30 estimators, split by entropy, max depth of five) was trained using 100-fold cross validation on single channel FDBB, TDBB, and EMC. The Gini Importance of each feature was aggregated from the pretrained models from each internal cross validation fold and external LOO fold, making a score distribution from 2400 importance calculations overall. Importance scores are represented in Fig. 2c.

### Deployment

Once the relationships between all features and their relative contributions to seizure detection were evaluated, we could develop ensembles of our black box and hand-crafted features from a completely new seizure dataset. This would prove the generalizability of our feature extractors and determine if our black-box features could be combined for downstream machine learning tasks.

#### Kaggle challenge

The Kaggle dataset was selected for evaluating the transferability of all feature ensembles because it: (i) has a relatively large and still private testing dataset, (ii) is graded and evaluated by a standardized third-party (the Kaggle auto grader), (iii) has two standardized and well-defined tasks (seizure and latency identification) that are completed by all other contestants, and (iv) Allows for a transparent comparison to other models through its public and private leaderboards, and (v) contains data from patients using completely different electrode configurations, and thus provides a clear way to test the generalizability of our model. Specifically, it contains approximately 7 min of training data with iEEG recordings from 4 canine and 8 human patients: the canine recordings were sampled with 16 implantable electrodes at 400 Hz^42^. Human subjects were recorded with varying electrode arrangements (30–72) using a scalable acquisition platform and were sampled using a 9 kHz anti-aliasing filter, 32 kHz sampling frequency, and a DC to 8 kHz bandpass filter. All human data was then down sampled to either 500 or 5000 Hz prior to release in the Kaggle competition^40,42,43^. The dataset also consisted of 549 min of test data from the same subjects with a seizure composition unknown to the public, all data was given in 1 s epochs. All human recordings were resampled to 400 Hz before analysis. The contest itself assigned contestants with designing two models: one for first identifying seizures, and another for determining if the seizure is within 15 s of onset, known as the seizure latency. All canine channels were referenced to the group average for each recording, and all human recordings were referenced to an electrode outside the brain^41^. An average of 35 electrodes were used per subject.

The deep neural network with the best AUC score on our seizure identification task was selected to attempt the Kaggle competition to allow us to compare the performance of our features to a traditional deep learning approach. Each subject had their own instance of the neural network module to prevent issues from mismatching channel counts. Each subject model was trained using stochastic gradient descent, Adam optimizer, and binary cross-entropy with five-fold cross validation by sample, where 25% of the validation set was held out for testing after each epoch. The TDBB was included to demonstrate the typical CNN paradigm of simultaneously learning spatial and temporal features from iEEG recordings, and thus provides a baseline for comparison; it was not used for feature selection.

The training set was augmented using a sliding window with 50% overlap to include all the “middle” time points between each second. The test set was used to generate the final response, and was graded by the Kaggle auto grader, which generated an ROC score representing the average score of the latency task and identification task.

All the LOO cross-validation folds from pretraining the TDBB and FDBB models were loaded and used to extract features from the Kaggle data, the engineered metrics were also extracted from the Kaggle data and concatenated with the TDBB and FDBB features. The features from each of the 25-folds were extracted and averaged together to generate the feature sets for the challenge. This is possible because each pretraining LOO fold has its own fully trained model, and there are 25 folds because each patient was held out once while pretraining the TDBB and FDBB models. From the averaged features, the three most significant and single most significant for both the identification and latency tasks were extracted from each channel, creating two separate feature sets tailored to each task (in this case, “significant” means high Gini Importance). The same was done with the least important features to serve as controls. All feature sets were used to train Random Forest classifiers for each subject (30 estimators, entropy, max depth of five) to evaluate the transferability of our DNNs and determine if they can be used for feature engineering. The trained estimator from each fold of this cross-validation step was used to create a Kaggle entry to allow for a distribution of 30 potential scores for various feature ensembles. It is important to emphasize that the neural networks used to extract the black box features were pretrained on data unrelated to the Kaggle set.

## Supplementary Information

Supplementary Figure 1.

## Data Availability

Data and code are available from the authors on reasonable request. The databases referenced by this project are: UPenn-Mayo Seizure Detection Challenge Kaggle Competition: https://www.kaggle.com/c/seizure-detection/overview/evaluation; ieeg.org: https://www.ieeg.org/; Epilepsiae: http://epilepsy-database.eu/.
