# Supplementary material for: Engineering nonlinear epileptic biomarkers using deep learning and Benford’s law
Source: Sci Rep. 2022 Mar 30;12:5397. doi: 10.1038/s41598-022-09429-w (PMC8967852; doi:10.1038/s41598-022-09429-w)
Supplement: Supplementary file 1 — Supplementary Figure 1. [file 41598_2022_9429_MOESM1_ESM.pdf]

Engineering Nonlinear Epileptic Biomarkers Using Deep Learning and Benford's Law

Joseph Caffarini MS[1], Klevest Gjini MD PhD[1], Brinda Sevak MS[1], Roger Waleffe BS[3], Mariel Kalkach-Aparicio MD[1], Melanie Boly MD PhD[1,2], Aaron F Struck MD[1,4]

Author affiliations:
[1]University of Wisconsin-Madison Department of Neurology
[2]University of Wisconsin-Madison Department of Psychiatry
[3]University of Wisconsin-Madison Department of Computer Science
[4]William S Middleton Memorial Veterans Hospital, Madison, WI

a.

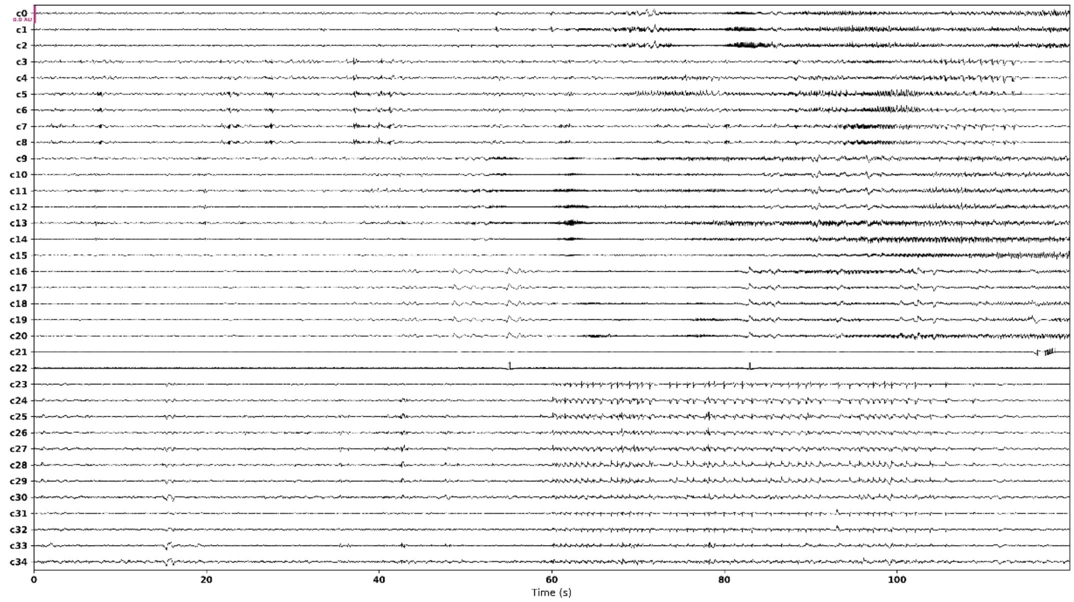
c0
c1
c2
c3
c4
c5
c6
c7
c8
c9
c10
c11
c12
c13
c14
c15
c16
c17
c18
c19
c20
c21
c22
c23
c24
c25
c26
c27
c28
c29
c30
c31
c32
c33
c34
0
20
40
60
80
100
Time (s)

b.

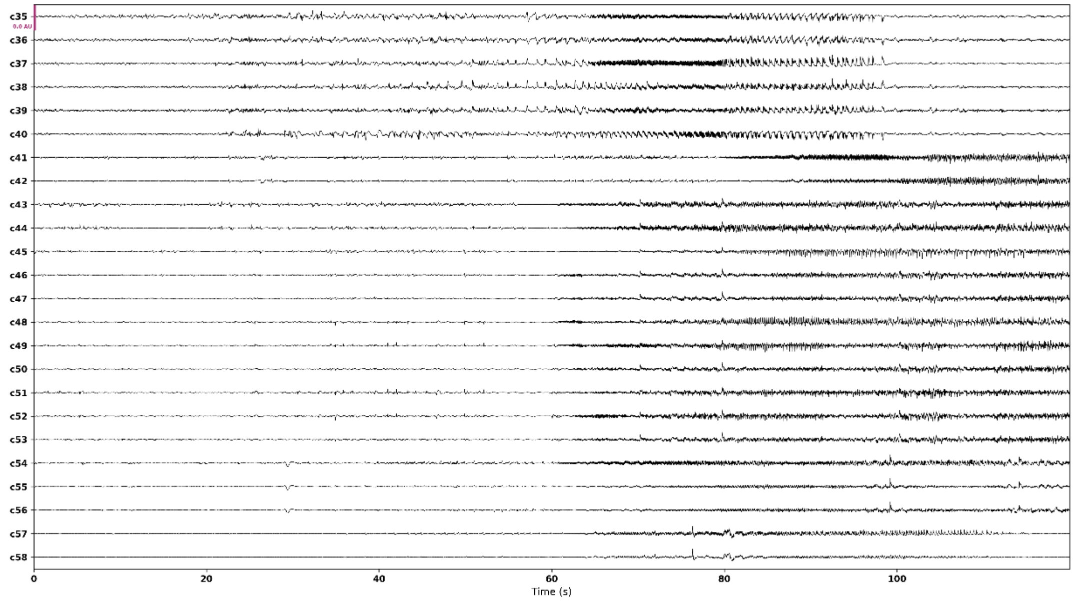
c35
c36
c37
c38
c39
c40
c41
c42
c43
c44
c45
c46
c47
c48
c49
c50
c51
c52
c53
c54
c55
c56
c57
c58
0
20
40
60
80
100
Time (s)

c.

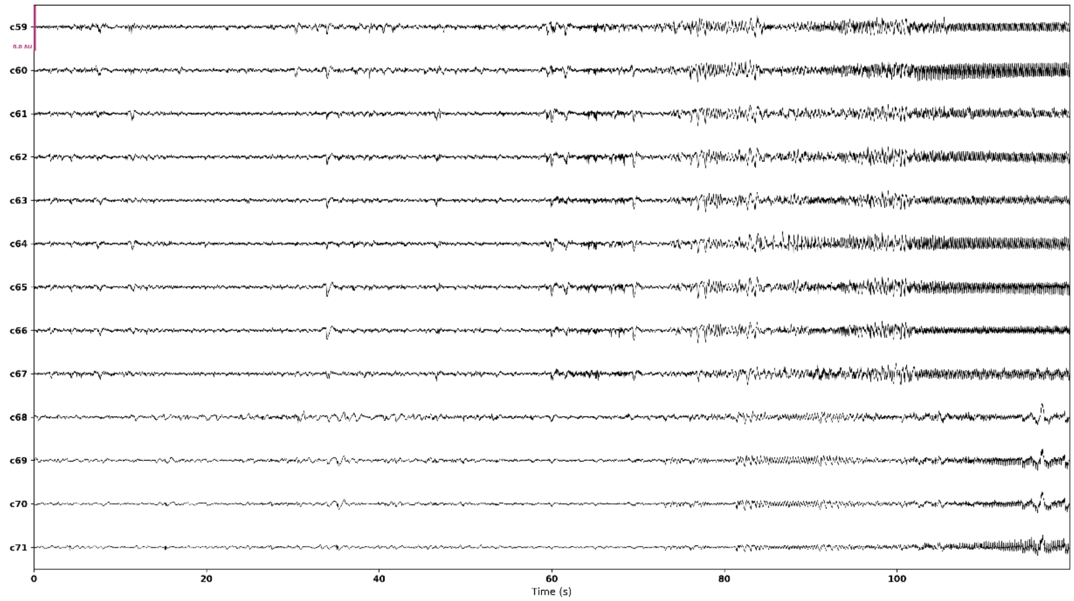
c59
c60
c61
c62
c63
c64
c65
c66
c67
c68
c69
c70
c71
0
20
40
60
80
100
Time (s)

d.

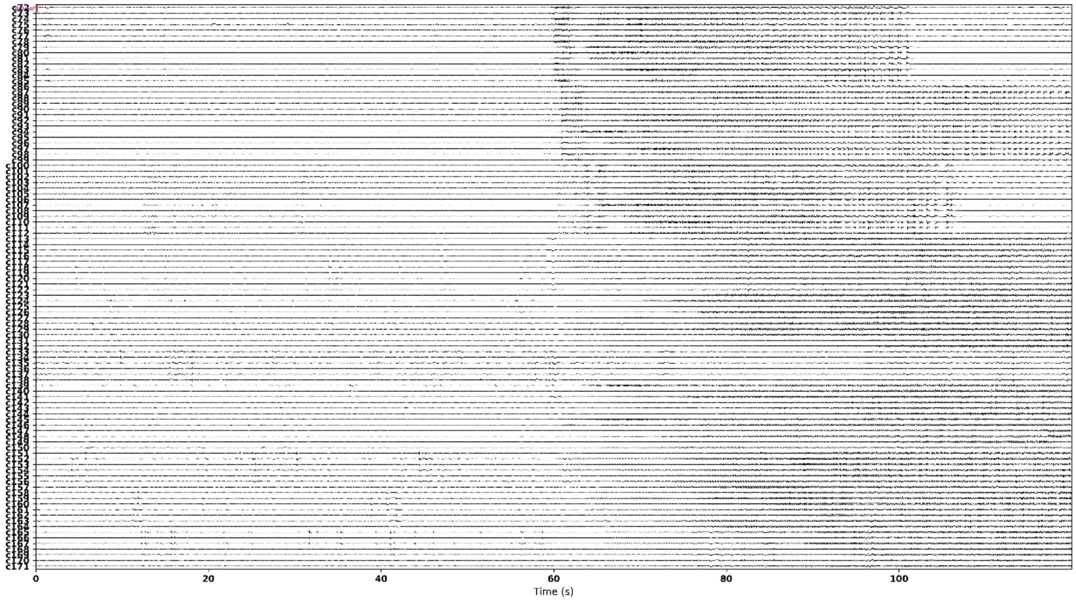
0
20
40
60
80
100
Time (s)

e.

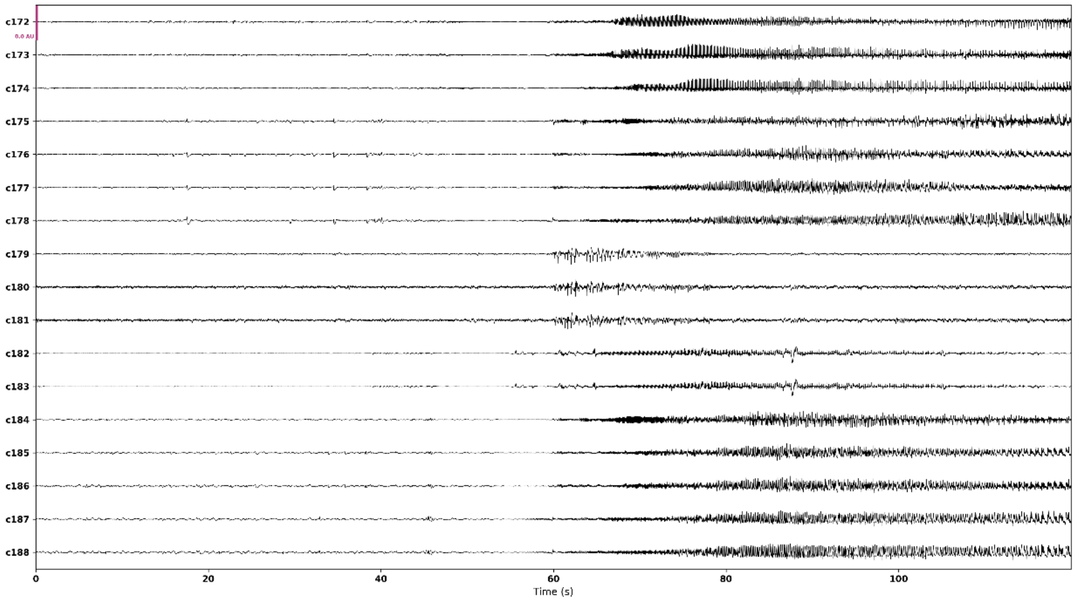
c172
c173
c174
c175
c176
c177
c178
c179
c180
c181
c182
c183
c184
c185
c186
c187
c188
0
20
40
60
80
100
Time (s)

f.

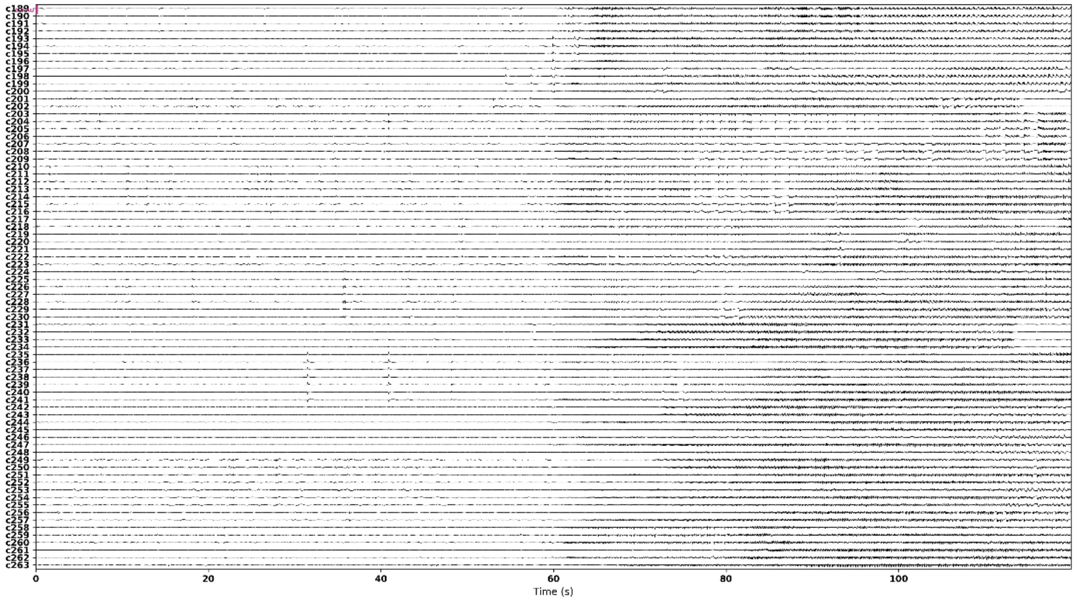
0
20
40
60
80
100
Time (s)

g.

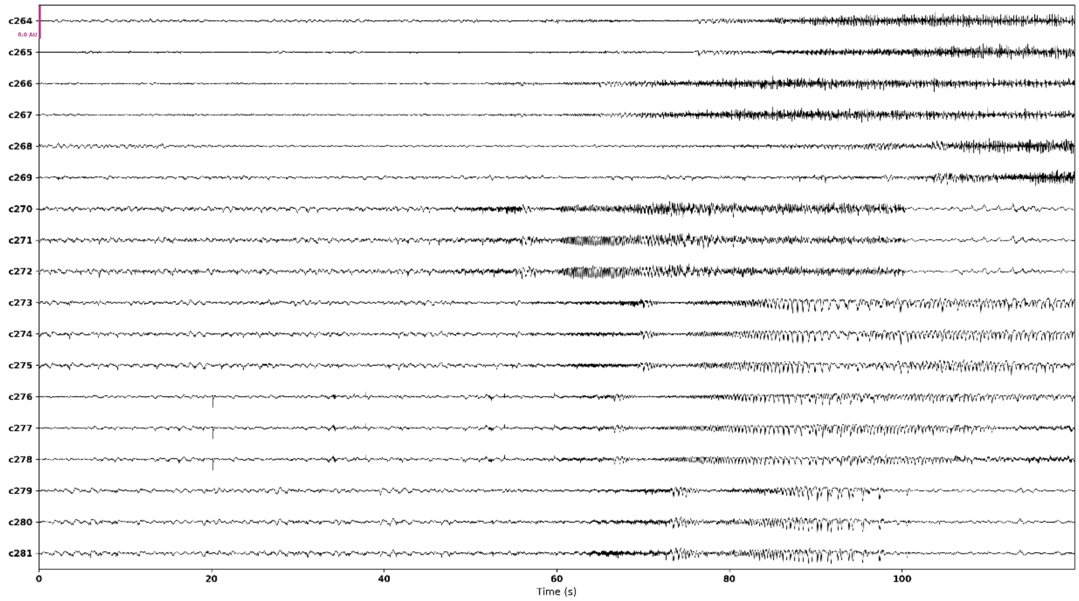
c264
0.0 AU
c265
c266
c267
c268
c269
c270
c271
c272
c273
c274
c275
c276
c277
c278
c279
c280
c281
0
20
40
60
80
100
Time (s)

h.

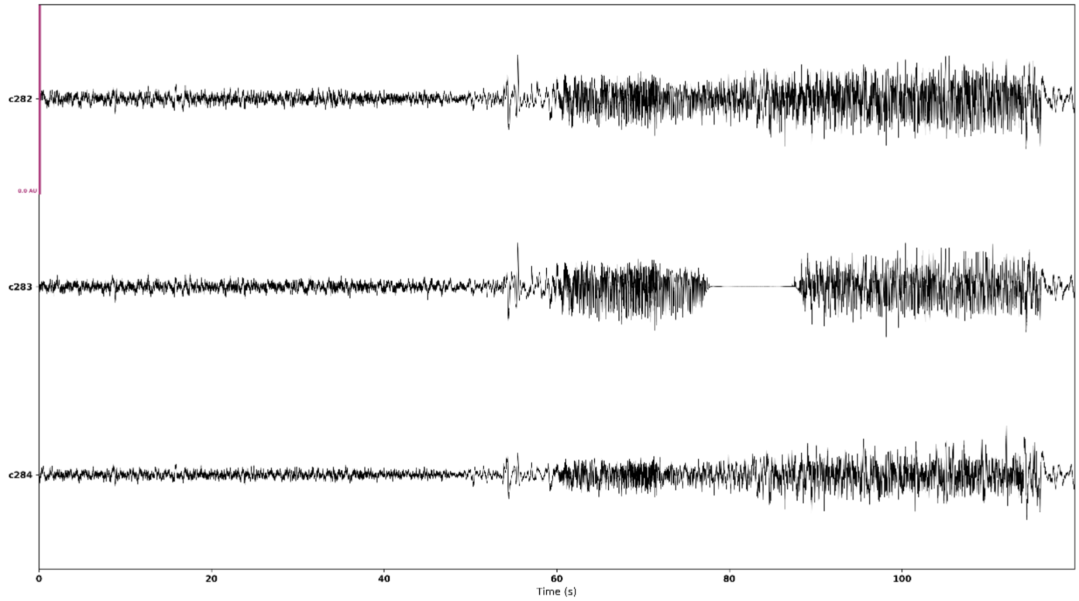
c282
0.0 AU
c283
c284
0
20
40
60
80
100
Time (s)

i.

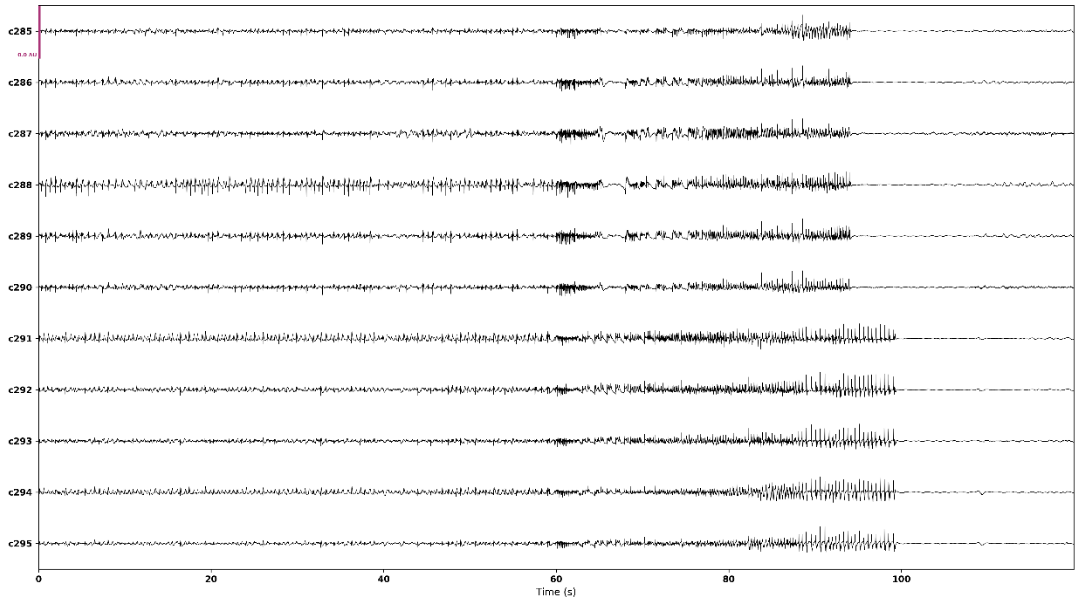
c285
c286
c287
c288
c289
c290
c291
c292
c293
c294
c295
0
20
40
60
80
100
Time (s)

j.

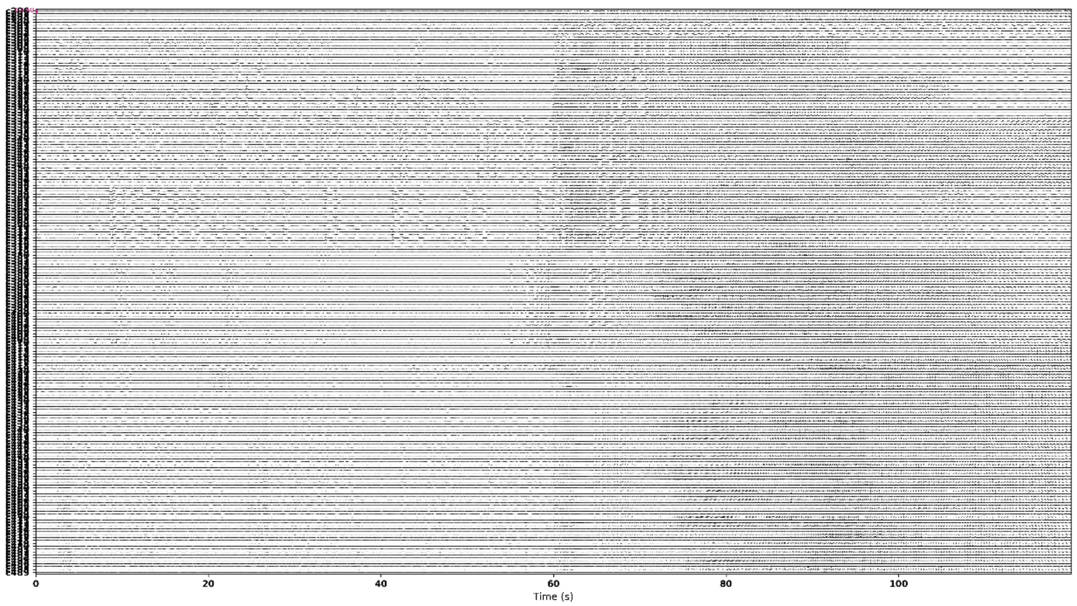
0
20
40
60
80
100
Time (s)

k.

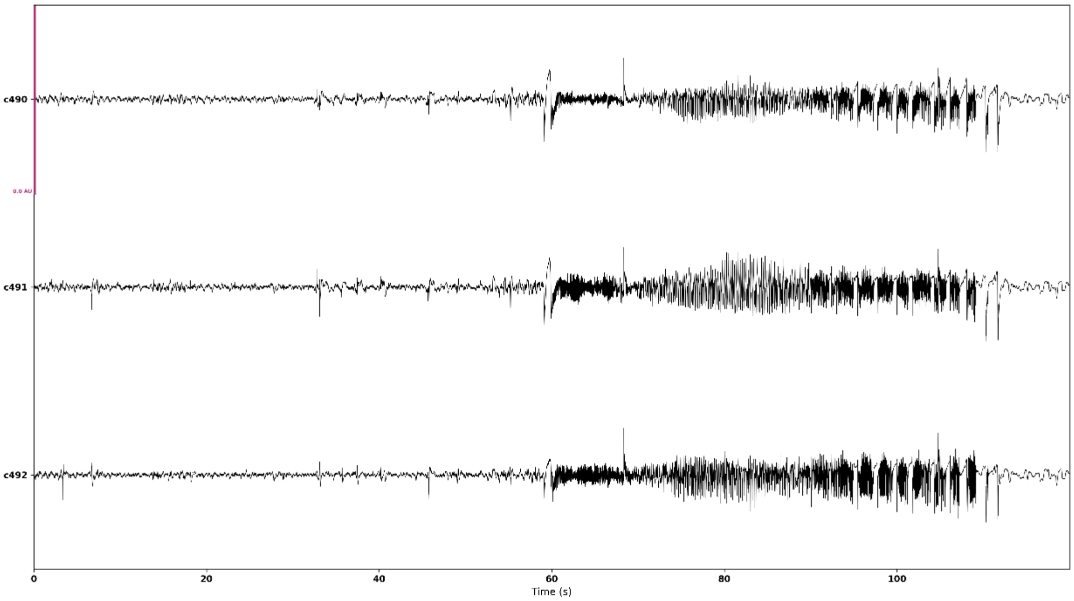
c490
0.0 AU
c491
c492
0
20
40
60
80
100
Time (s)

l.

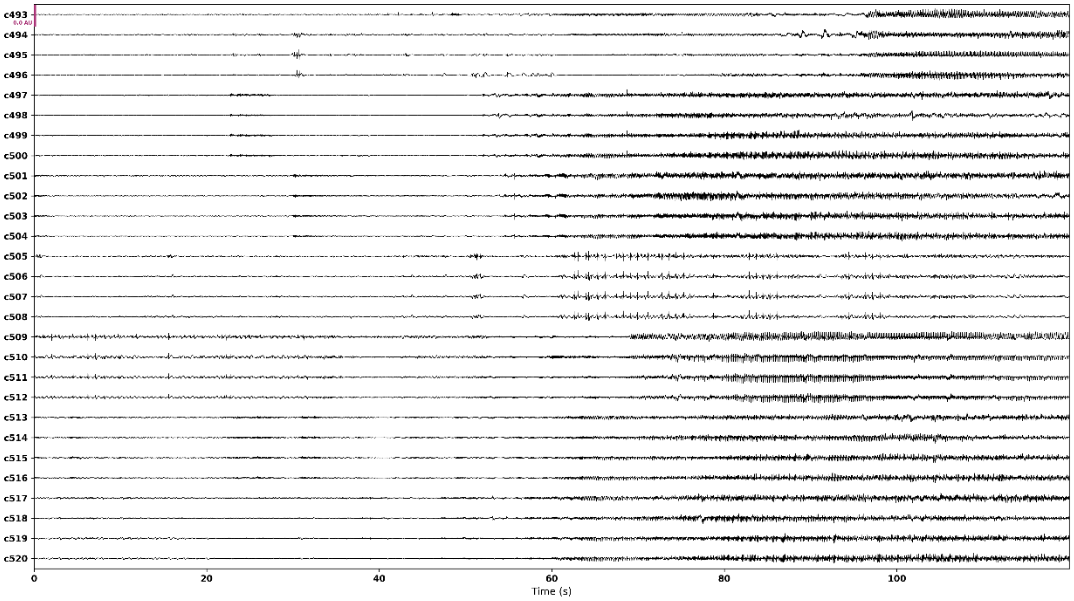
c493
0.0 AU
c494
c495
c496
c497
c498
c499
c500
c501
c502
c503
c504
c505
c506
c507
c508
c509
c510
c511
c512
c513
c514
c515
c516
c517
c518
c519
c520
0
20
40
60
80
100
Time (s)

m.

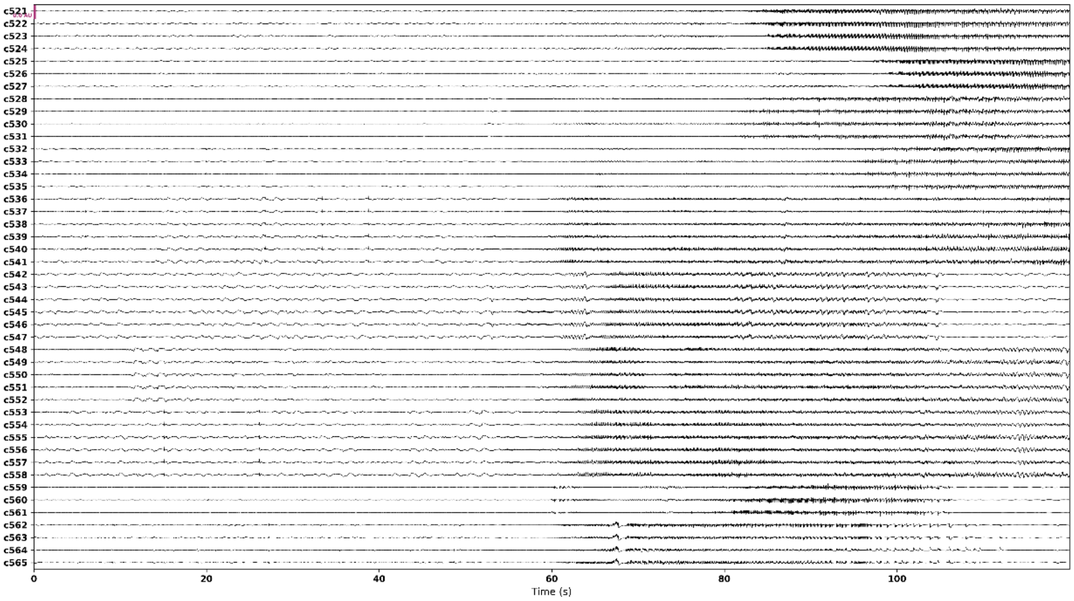
c521
c522
c523
c524
c525
c526
c527
c528
c529
c530
c531
c532
c533
c534
c535
c536
c537
c538
c539
c540
c541
c542
c543
c544
c545
c546
c547
c548
c549
c550
c551
c552
c553
c554
c555
c556
c557
c558
c559
c560
c561
c562
c563
c564
c565
0
20
40
60
80
100
Time (s)

n.

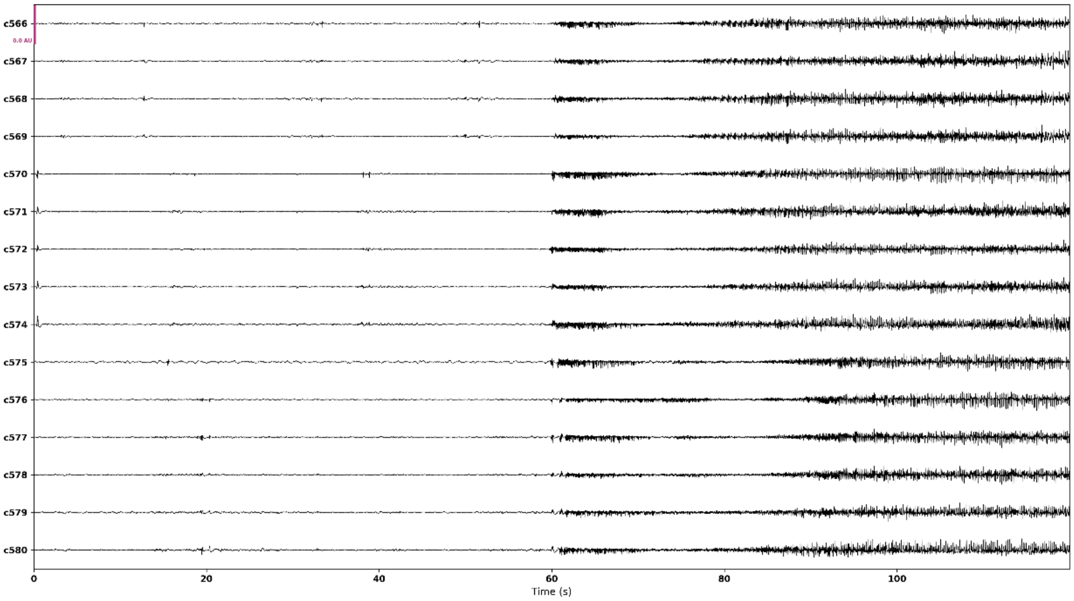
c566
0.0 AU
c567
c568
c569
c570
c571
c572
c573
c574
c575
c576
c577
c578
c579
c580
0
20
40
60
80
100
Time (s)

o.

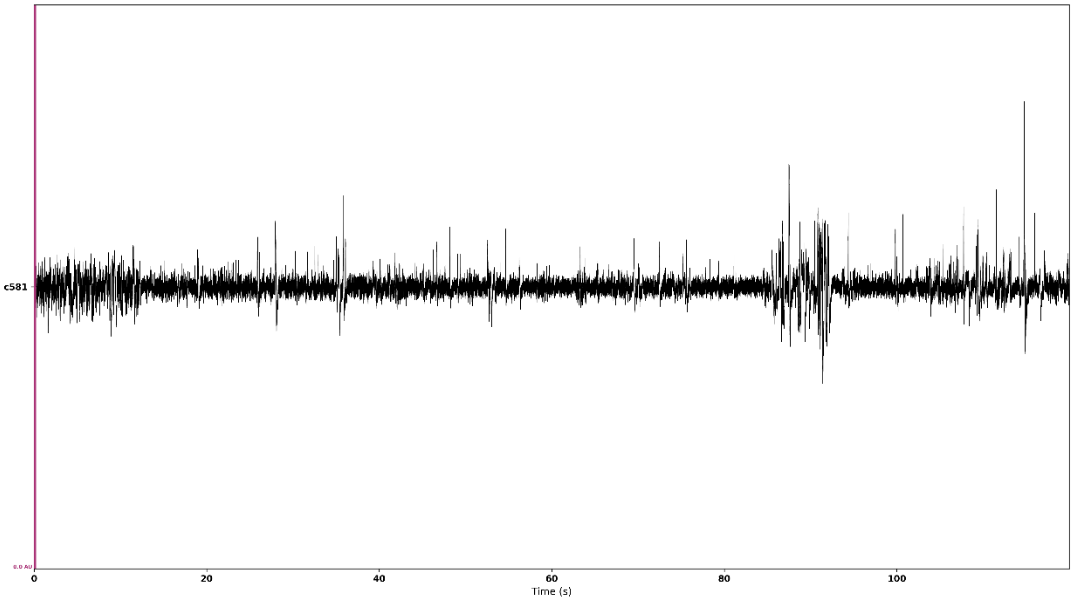

c581
0.0 AU
0
20
40
60
80
100
Time (s)

p.

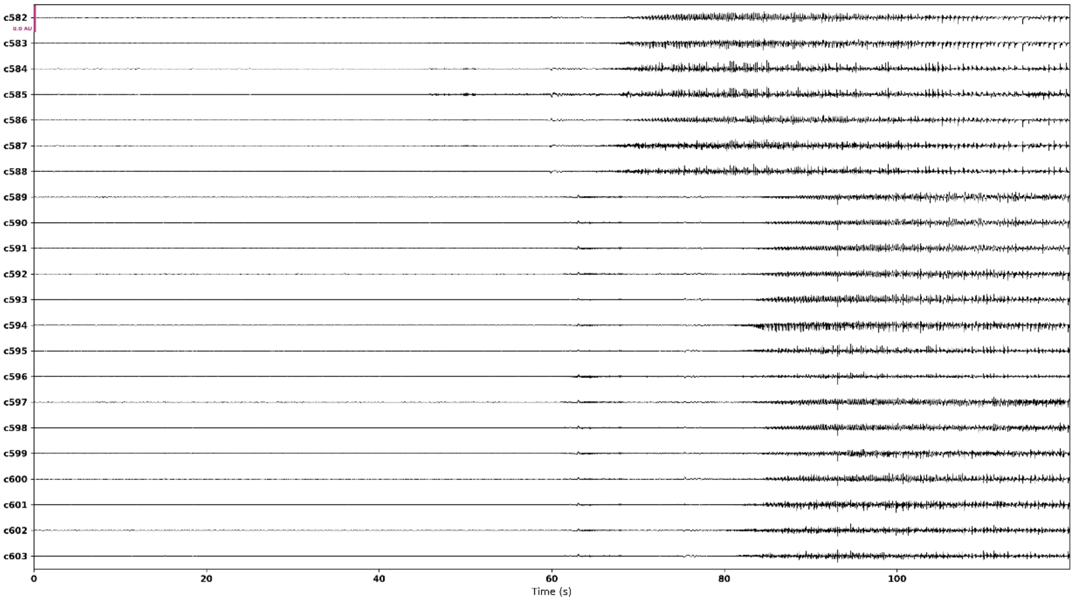

c582
c583
c584
c585
c586
c587
c588
c589
c590
c591
c592
c593
c594
c595
c596
c597
c598
c599
c600
c601
c602
c603
0.0 AU
0
20
40
60
80
100
Time (s)

q.

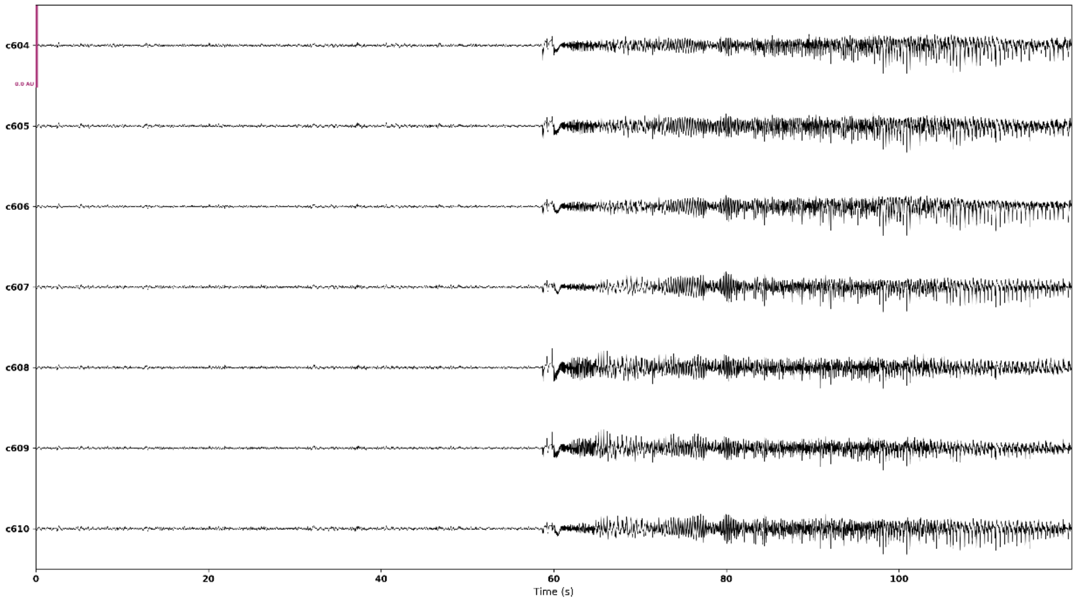
c604
c605
c606
c607
c608
c609
c610
0.0 AU
0
20
40
60
80
100
Time (s)

r.

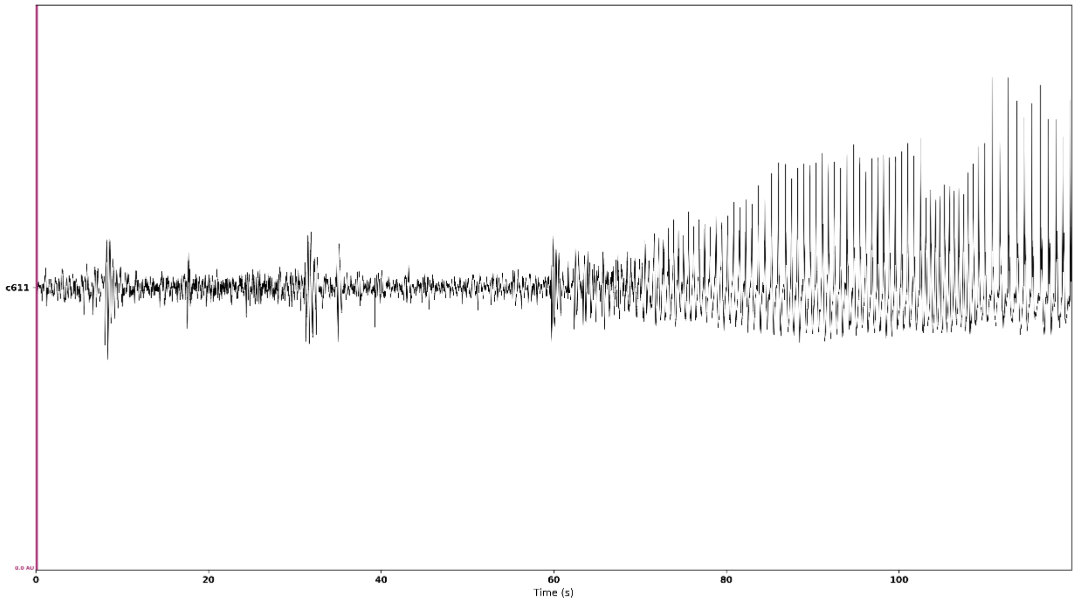
c611
0.0 AU
0
20
40
60
80
100
Time (s)

s.

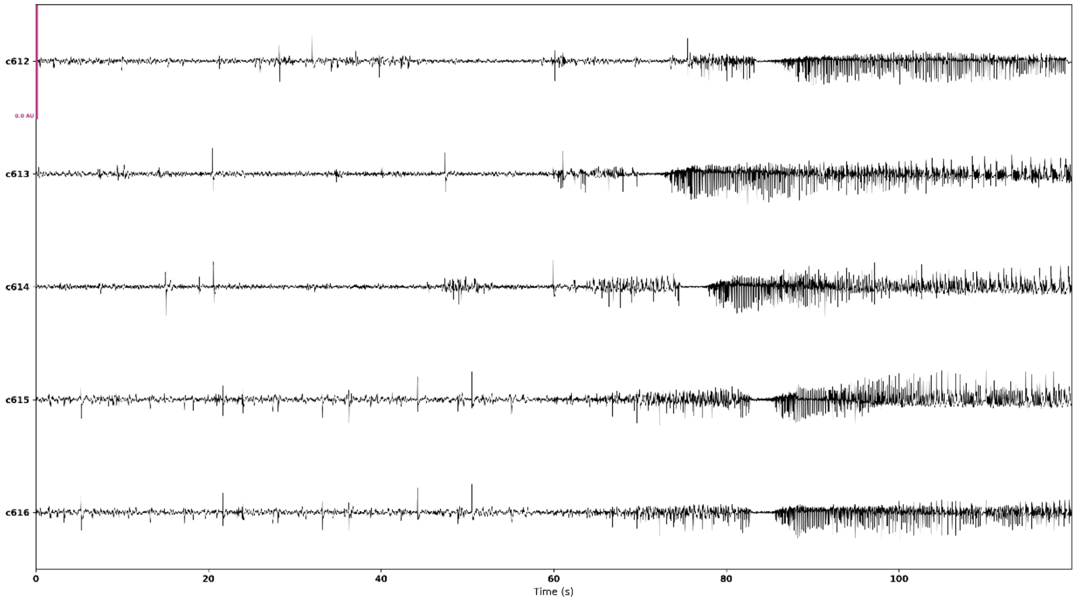

c612
0.0 AU
c613
c614
c615
c616
0
20
40
60
80
100
Time (s)

t.

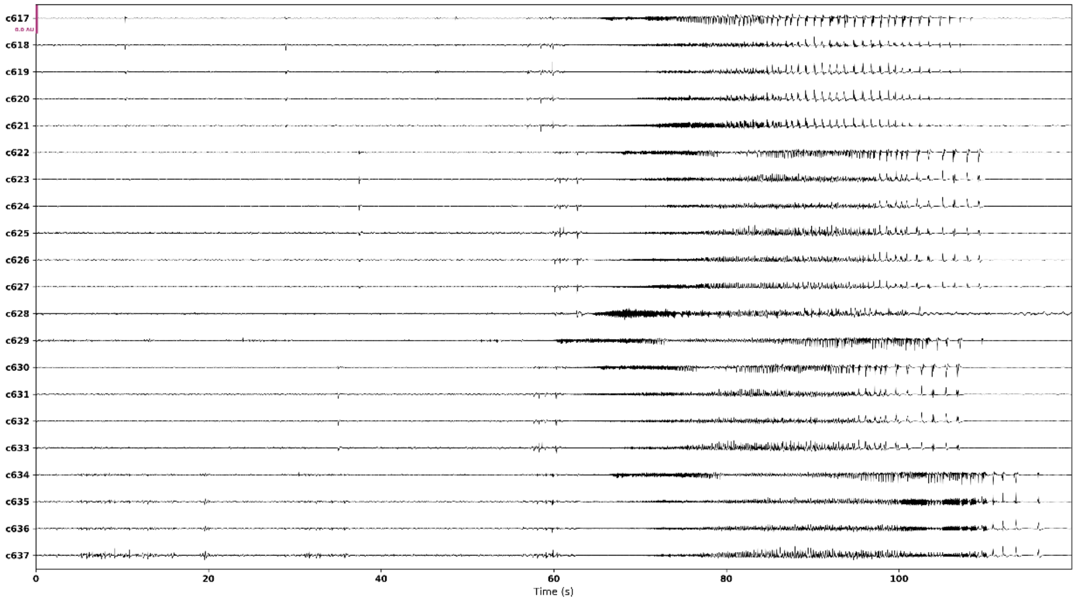

c617
c618
c619
c620
c621
c622
c623
c624
c625
c626
c627
c628
c629
c630
c631
c632
c633
c634
c635
c636
c637
0
20
40
60
80
100
Time (s)

u.

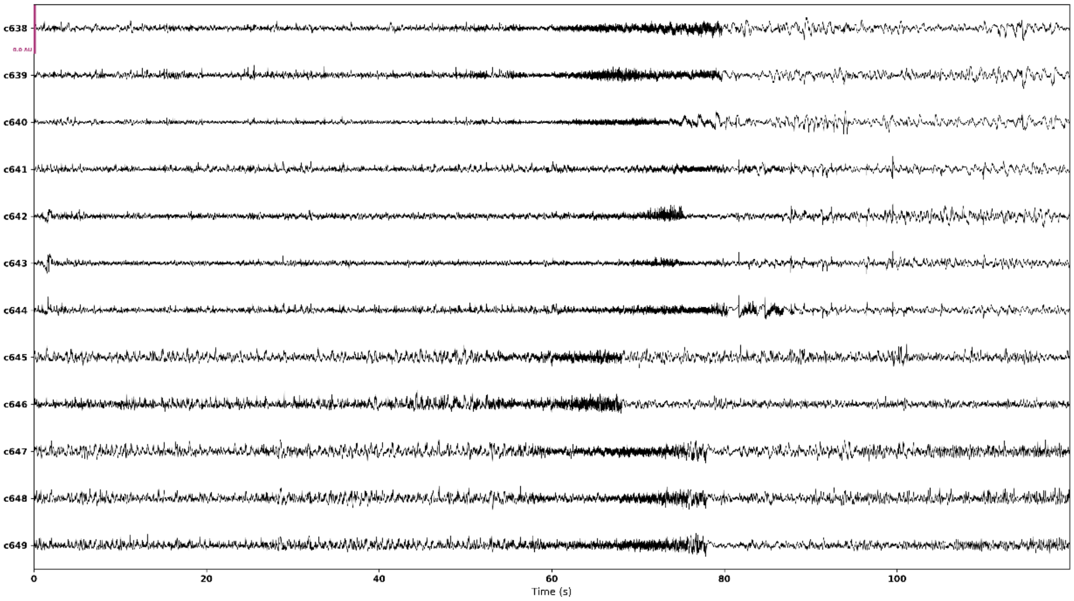
c638
0.0 AU
c639
c640
c641
c642
c643
c644
c645
c646
c647
c648
c649
0
20
40
60
80
100
Time (s)

v.

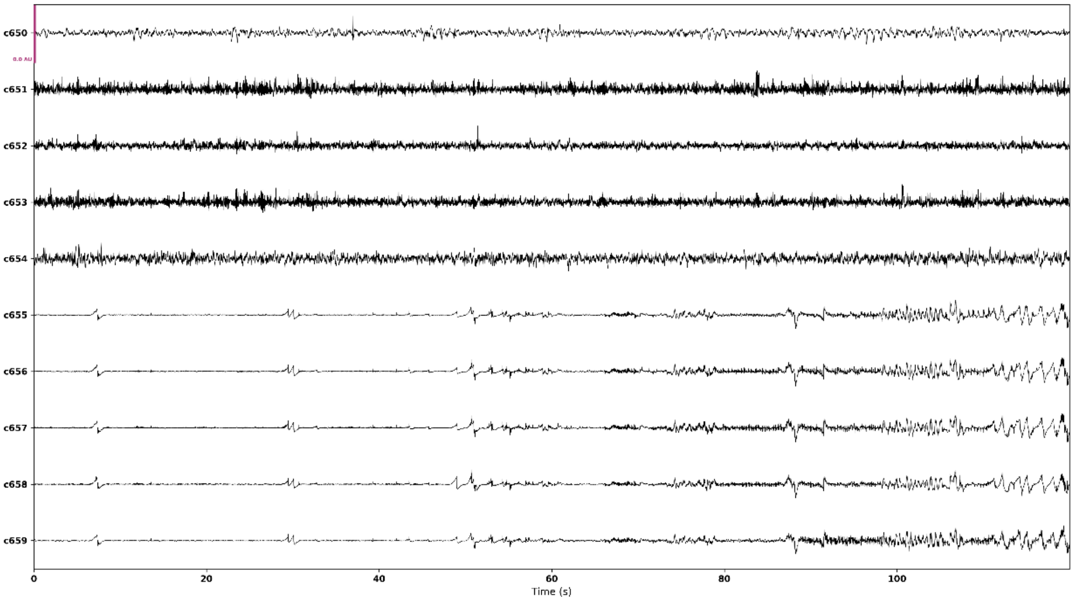
c650
0.0 AU
c651
c652
c653
c654
c655
c656
c657
c658
c659
0
20
40
60
80
100
Time (s)

w.

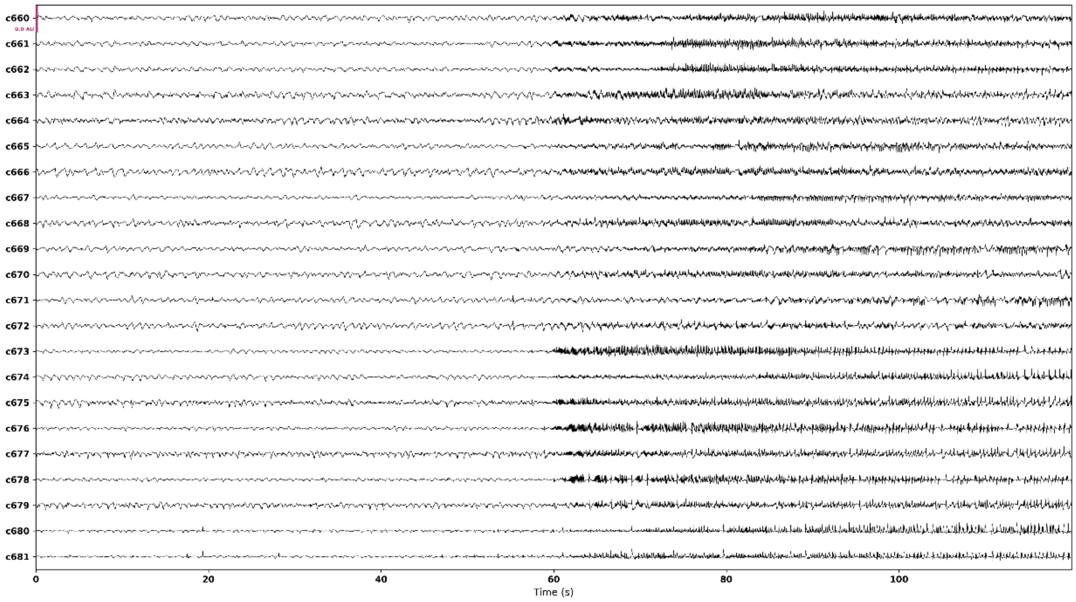
c660
0.0 AU
c661
c662
c663
c664
c665
c666
c667
c668
c669
c670
c671
c672
c673
c674
c675
c676
c677
c678
c679
c680
c681
0
20
40
60
80
100
Time (s)

x.

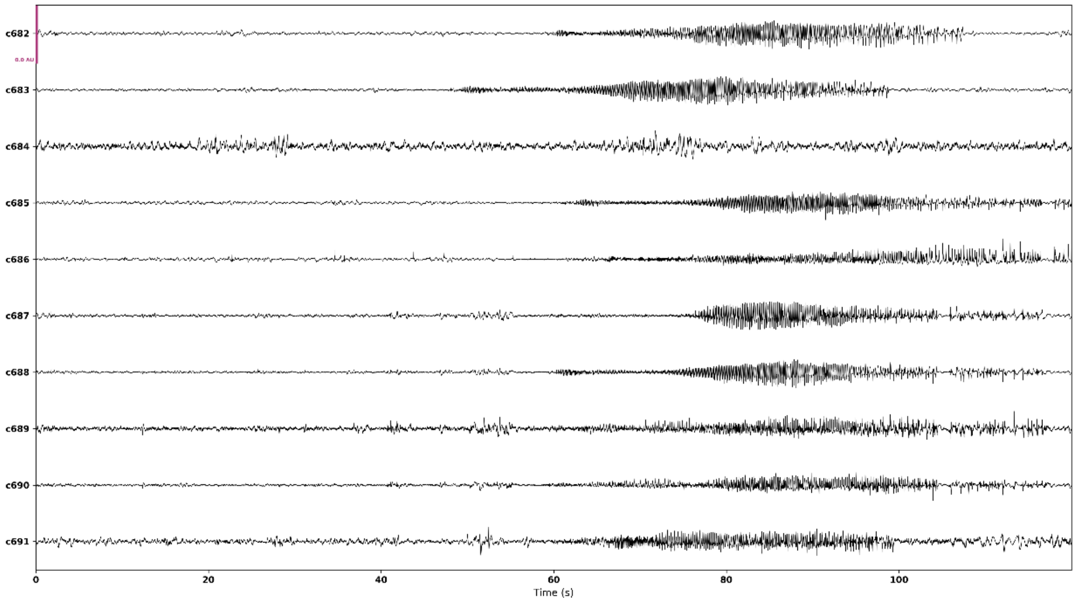
c682
0.0 AU
c683
c684
c685
c686
c687
c688
c689
c690
c691
0
20
40
60
80
100
Time (s)

y.

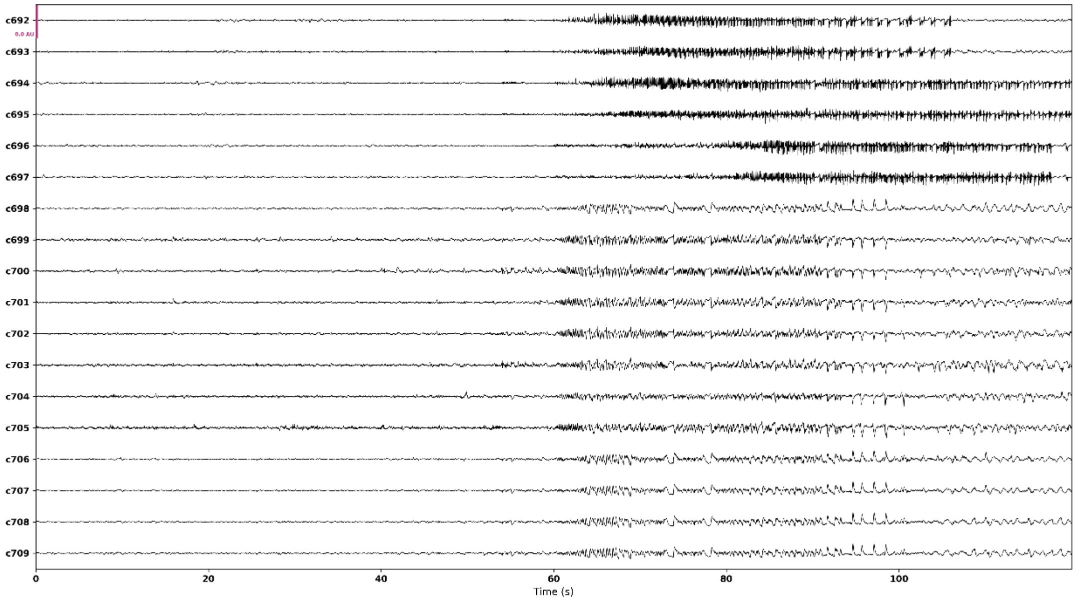

c692
0.0 AU
c693
c694
c695
c696
c697
c698
c699
c700
c701
c702
c703
c704
c705
c706
c707
c708
c709
0
20
40
60
80
100
Time (s)

z.

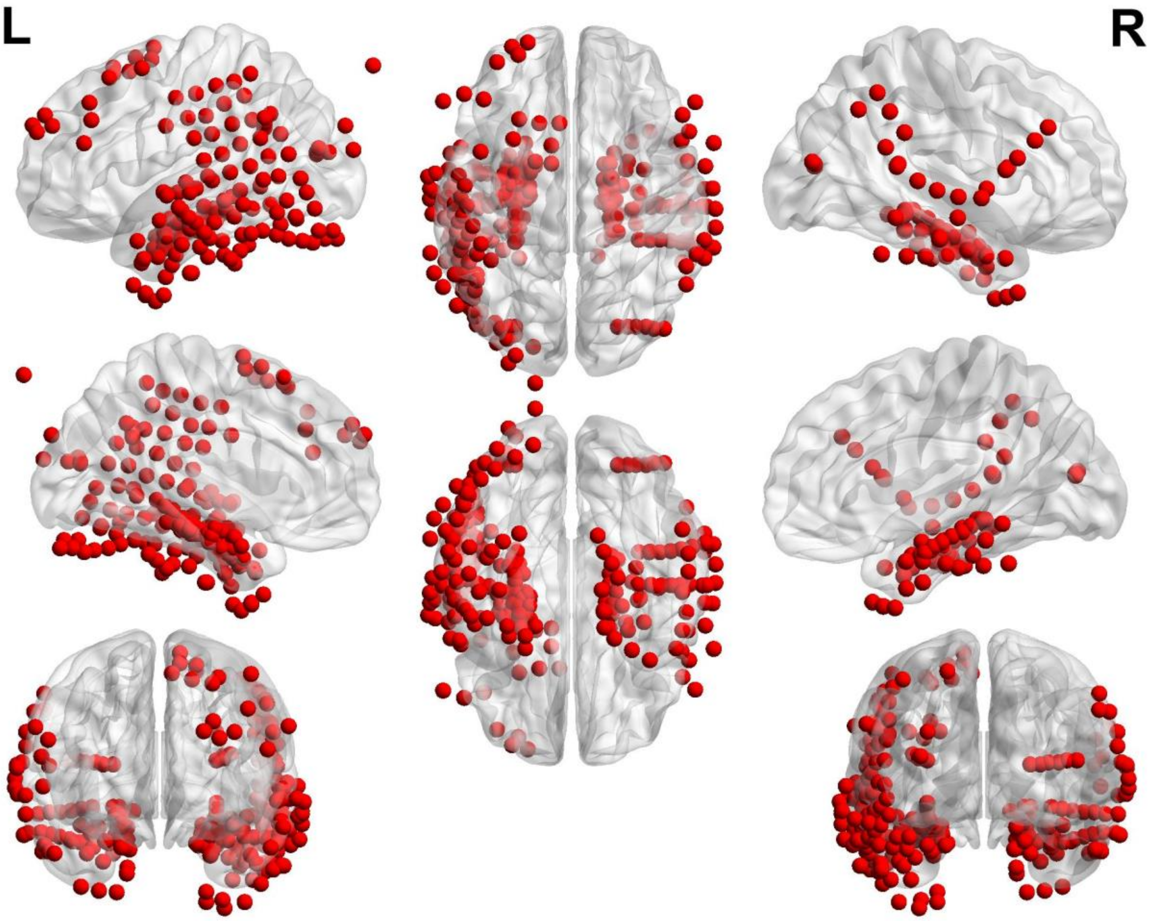

L
R

Figure 1: iEEG Recordings used for pretraining neural networks, separated by Leave-One-Out test folds. a-y) IEEG recordings are ordered as they appear in each row of **Figure 1d**. Each one of these figures served as the LOO test set at some point, making 25 separate folds. Another subset of 8 subjects was used as the validation set within each fold, further preventing data leakage. All recordings were cropped so that all seizures begin at 60 seconds into the recording. **z) Brain regions involved in pretraining dataset.** Shown are all the separate electrodes from each subject summarized on the same anatomy to show the brain regions included in the pretraining task.
